# Non-perturbative treatment of the solid effect of dynamic nuclear polarization

**DOI:** 10.5194/mr-4-129-2023

**Published:** 2023-06-05

**Authors:** Deniz Sezer

**Affiliations:** Institute of Physical and Theoretical Chemistry, Goethe University, 60438 Frankfurt am Main, Germany

## Abstract

In the solid effect of dynamic nuclear polarization (DNP), the concerted flips of the electronic and nuclear spins, which are needed for polarization transfer, are induced by the microwaves. Commonly, the effect of the microwaves is modeled by a rate process whose rate constant is determined perturbatively. According to quantum mechanics, however, the coherent microwave excitation leads to Rabi nutation, which corresponds to a rotation rather than a rate process. Here we reconcile the coherent effect of the microwaves with the description by rate equations by focusing only on the steady state of the spin dynamics. We show that the phenomenological rate constants describing the synchronous excitation of the electronic and nuclear spins can be selected such that the description by rate equations yields the same steady state as the exact quantum-mechanical treatment. The resulting non-perturbative rates differ from the classical, perturbative ones and remain valid also at the high microwave powers used in modern-day DNP. Our treatment of the solid effect highlights the role of the coherences in the mechanistic steps of polarization transfer and reveals the importance of the dispersive (i.e., out-of-phase) component of the EPR line. Interestingly, the multiplicative dependence of the DNP enhancement on the dispersive EPR component was intuited in the very first report of the solid effect in liquids [Bibr bib1.bibx13]. The time-domain description of the solid effect developed here is extendable to liquids, where the dipolar interaction changes randomly in time due to molecular diffusion.

## 1 Introduction

The Boltzmann polarization of electronic spins in a magnetic field is orders of magnitude larger than that of nuclear spins. When the electronic and nuclear spins interact with each other, it becomes possible to transfer the much larger polarization of the former to the latter. Such transfer, known as dynamic nuclear polarization (DNP), can be achieved in several ways, which differ in their mechanistic steps. Two of the DNP mechanisms, namely the Overhauser effect and the solid effect, can be explained by considering a minimal system comprising one electronic spin and one nuclear spin. To explain the other two DNP mechanisms known as the cross effect and thermal mixing, it is necessary to consider one nuclear spin interacting with, respectively, two and many coupled electronic spins [Bibr bib1.bibx33]. The current paper engages only with the former two DNP mechanisms.

Historically, the Overhauser effect was the first to be conceived [Bibr bib1.bibx22] and observed experimentally, initially in metals and subsequently also in liquids [Bibr bib1.bibx8]. A rigorous theoretical understanding of the effect in nonmetals was provided shortly after the first experiments [Bibr bib1.bibx1]. At the core of this understanding are the Solomon equations, which describe the relaxation processes in a system of two interacting spins [Bibr bib1.bibx31]. For our purposes, it is useful to discern two aspects of the theoretical formalism. On the one hand, the evolution of the electronic and nuclear polarizations is described by two coupled differential equations [Bibr bib1.bibx31], analogous to the rate equations of chemical kinetics. On the other, the phenomenological rate constants that appear in these rate equations are expressed in terms of the quantum-mechanical probabilities for transition between two distinct energy states [Bibr bib1.bibx31]. To first order in a perturbative calculation, the amplitude of such transition probability per unit time is proportional to the matrix element of the relevant interaction term in the spin Hamiltonian [Bibr bib1.bibx31]. While the name Solomon equations is mainly used to refer to the first of these aspects [Bibr bib1.bibx19], the perturbative calculation of the transition probabilities per unit time is an integral part of the theoretical description. In fact, the idea that interaction terms in the Hamiltonian have corresponding probabilities per unit time to induce transitions (i.e., what we have called the second aspect of the theory) provides the logical justification for the description by rate equations [Bibr bib1.bibx1].

The solid-state effect (or solid effect) was the second DNP effect to be observed experimentally and explained theoretically [Bibr bib1.bibx4]. In the Overhauser effect, the simultaneous flips of the electronic and nuclear spins, which are needed to couple the electronic and nuclear polarizations, are achieved by thermal relaxation; in the solid effect, these synchronous spin flips are driven coherently by the microwave irradiation. Thus, in the solid effect, the phenomenological rate constants of the rate equations are calculated from the matrix elements of the microwave term in the Hamiltonian. For this term to excite nuclear spin flips, the dipolar interaction between the electronic and nuclear spins should mix the Zeeman energy states and thus make the zero-quantum (ZQ) and double-quantum (DQ) transitions weakly allowed [Bibr bib1.bibx4].

Although the Overhauser effect and the solid effect are described using a consistent theoretical formalism (with its two complementary aspects explained above), quantum-mechanically there is a major difference between relaxation and coherent excitation. By their very nature, the rate equations of the polarizations model all evolution as exponential decay/increase towards some steady state. However, according to quantum mechanics, the effect of the microwave field is to rotate the magnetization, leading to the phenomenon known as Rabi nutation. Since rotation and exponential decay/increase are fundamentally different, modeling the effect of the microwaves as a relaxation process should not be possible in general. This raises questions about the fundamental applicability of the first aspect of our theoretical understanding, namely the rate-equation formalism, to the description of the coherently driven polarization transfer in the solid effect (as opposed to the relaxation-driven transfer in the Overhauser effect). Because the rate equations are justified by the idea that interaction terms induce transitions with a constant probability per unit time, the possibility to model the effect of the microwaves through a perturbative rate constant also becomes questionable. It should be pointed out that these concerns are not new. Indeed, in the case of single spin 1/2 , where the quantum dynamics is described exactly by the Bloch equations, Abragam explicitly analyzes how the rate equation with a perturbative rate constant for the microwave (mw) excitation relates to the exact solution, both at short times and at long times pp. 27–32.

While many modern applications of DNP in the solid state rely on pulsed methods [Bibr bib1.bibx7], here we consider only continuous-wave (cw) excitation, where one is exclusively interested in the steady state of the spin dynamics. As a result, we will be only concerned with how the description of the mw excitation by rate equations relates to the steady state of the proper quantum-mechanical description. To this end, in Sect. [Sec Ch1.S2] we examine the two descriptions for a single spin 1/2 and, following [Bibr bib1.bibx2], confirm that the perturbative rate constant of mw excitation leads to the same steady state as the Bloch equations.

Motivated by this observation, in Sect. [Sec Ch1.S3] we adopt the same perspective to analyze the system composed of one electronic and one nuclear spin 1/2 . In this case, starting with the Liouville–von Neumann equation of the density matrix, we first derive proper quantum-mechanical equations of motion for the expectation values of the spin operators that are relevant to the solid effect. Then we show that one can analytically solve for the steady state of the exact quantum dynamics, under the simplifying assumption that the dynamics of the electronic spins is not affected by the hyperfine interaction with the nuclei. Since, at steady state, all coherences can be expressed in terms of the polarizations, it becomes possible to rewrite the dynamical equations in terms of the polarizations only. Comparing the resulting equations with the rate equations of the polarizations, we select the phenomenological rate constants that appear in the latter, such that the two descriptions have identical steady states.

Stated differently, we abolish the idea of constant transition probabilities per unit time as justification for the rate equations. Instead, we view the rate equations as a convenient mnemonic for encoding the steady state of the exact quantum dynamics. Having decoupled the phenomenological rate constants from the perturbative calculation of the mw-induced transition probabilities, we are free to select them such that the mnemonic yields the correct steady state, thus providing a shortcut to its analysis. We find that the rate constants for the ZQ and DQ transitions selected in this way differ from the corresponding perturbative rate constants that are currently used in the literature [Bibr bib1.bibx3].

In Sect. [Sec Ch1.S6] we show that our new rate constants reproduce the classical expressions when the Rabi nutation frequency $\omega_{1}$ is much smaller than the nuclear Larmor frequency $\omega_{I}$ , as required by the perturbative treatment. Our new analytical expressions for driving the forbidden transitions, however, also hold when $\omega_{1}>\omega_{I}$ , as could happen at S and X bands, given the high microwave powers currently employed in DNP experiments with resonance structure [Bibr bib1.bibx21]. These new expressions are the main analytical result of the current paper.

A complete description of the spin dynamics of the four-level system that we analyze here requires only 16 different spin operators, including the identity operator. The dynamics is thus encoded by a 16×16 propagation matrix in Liouville space and can be simulated numerically using a spin-dynamics simulation package [Bibr bib1.bibx6]. Such numerical simulations are currently often employed to explore the efficiency of the solid effect for various experimental parameters. However, even in the relatively simple case of a four-level system, observing a certain effect in the simulations does not automatically provide understanding about the mechanism of this effect, as demonstrated recently by [Bibr bib1.bibx25], who strive to explain the origin of a dispersive DNP component seen both in experiments [Bibr bib1.bibx29] and in their numerical simulations. Clearly, developing intuition about the spin dynamics that is relevant for a given phenomenon is invaluable.

The general quantum dynamics of a four-level system can be described through 15 coupled differential equations for the expectation values of the 15 spin operators, excluding the identity. The equations that we derive in Sect. [Sec Ch1.S3.SS2], together with the Bloch equations from Sect. [Sec Ch1.S2.SS2], constitute seven such equations. (In fact, we implicitly account for three more operators, thus covering 10 out of the 15 possible ones, as explained in Sect. [Sec Ch1.S4].) When the number of coupled differential equations increases beyond three, gaining an intuitive insight into the dynamics that they describe becomes difficult.

Inspired by the graphical representation of chemical reactions in biochemistry, in Sect. [Sec Ch1.S4] we represent visually the coupled differential equations describing the solid-effect spin dynamics. The resulting “flow diagram” sheds light on the dynamical interconnections between the spin polarizations and the coherences that are active in the solid effect. In Sect. [Sec Ch1.S5] we study the algebraic relationships between the coherences and the polarizations that emerge at steady state. When considered in the context of the dynamical interconnections, these algebraic relationships highlight the importance of the purely electronic coherences in the transfer of polarization, with the out-of-phase (i.e., dispersive) component playing a prominent role. Interestingly, the importance of the dispersive EPR line for the solid effect was intuited already in the first report of the solid effect in liquids [Bibr bib1.bibx13], as we discuss in Sect. [Sec Ch1.S7.SS2]. Our conclusions are presented in Sect. [Sec Ch1.S8].

## 2 Allowed EPR transition

In the rate-equation treatment of the Overhauser and solid effects [Bibr bib1.bibx32], both thermal relaxation and mw excitation are envisioned as randomly flipping spins between pairs of energy levels with certain rates, as depicted in Fig. [Fig Ch1.F1]. The current section aims to illustrate the analytical strategy that we will employ to analyze the solid effect, in the simplest possible case of a single spin 1/2 (Fig. [Fig Ch1.F1]a). We first present the rate equation of the electronic polarization and obtain its steady state (Sect. [Sec Ch1.S2.SS1]). Then we turn to the Bloch equations and also obtain their steady state (Sect. [Sec Ch1.S2.SS2]). Finally, by requiring that the two descriptions have identical steady states, we identify the rate constant that should be used to describe the effect of the microwaves in the phenomenological rate equation.

### 2.1 Rate equation of the electronic polarization

Let $n_{+}$ and $n_{-}$ be the populations of the two energy levels in Fig. [Fig Ch1.F1]a. Assuming the spins are not destroyed or created, the sum of the two populations is constant in time. Treating the mw excitation as a process that randomly flips the spins with rate constant $v_{1}$ , we have

1 $${\dot{n}}_{+}{|}_{\mathrm{mw}}=-{\dot{n}}_{-}{|}_{\mathrm{mw}}=-v_{1}\left(n_{+}-n_{-}\right).$$

(The subscript of the vertical bar indicates that the time derivative accounts only for mw excitation.) Note that $v_{1}\geq 0$ , since a negative rate constant does not make physical sense.

The electronic spin polarization $P_{S}=(n_{+}-n_{-})/(n_{+}+n_{-})$ is negative at thermal equilibrium, i.e., $P_{S}^{\mathrm{eq}}<0$ . Differentiating $P_{S}$ with respect to time and using Eq. ([Disp-formula Ch1.E1]), we find ${\dot{P}}_{S}{|}_{\mathrm{mw}}=-2v_{1}P_{S}$ for the effect of the mw irradiation. The action of thermal relaxation is analogous, after replacing $v_{1}$ by $w_{1S}$ and taking into consideration that $P_{S}$ decays towards its thermal equilibrium: ${\dot{P}}_{S}{|}_{\mathrm{th}}=-2w_{1S}(P_{S}-P_{S}^{\mathrm{eq}})$ . Combining the contributions of mw excitation and thermal relaxation, we get

2 $${\dot{P}}_{S}=-2v_{1}P_{S}-R_{1S}\left(P_{S}-P_{S}^{\mathrm{eq}}\right),$$

where $R_{1S}=2w_{1S}$ . The electronic longitudinal relaxation time is $T_{1S}=1/R_{1S}$ .

In the case of cw irradiation, one is interested in the steady state of the electronic polarization. When the left-hand side of Eq. ([Disp-formula Ch1.E2]) is set equal to zero,

3 $$P_{S}^{\mathrm{ss}}=\frac{R_{1S}}{R_{1S}+2v_{1}}P_{S}^{\mathrm{eq}}=pP_{S}^{\mathrm{eq}},$$

where the second equality defines the factor p . We refer to p as the electronic polarization factor, since it quantifies how close the steady-state polarization is to its Boltzmann value.

The rate equation (Eq. [Disp-formula Ch1.E2]) models the competition between mw pumping and the (longitudinal) relaxation of the polarization. When the two effects balance each other, the polarization is given by the steady-state solution (Eq. [Disp-formula Ch1.E3]). For the rate equation to be a predictive tool, it is necessary to express the phenomenological rate constants $v_{1}$ and $w_{1S}$ in terms of more fundamental quantities. As discussed in the Introduction, these are identified with the probabilities of transition per unit time between the two energy levels (Fig. [Fig Ch1.F1]a), which are calculated from time-dependent perturbation theory to first order [Bibr bib1.bibx31]. In the case of $v_{1}$ , this is basically Fermi's golden rule chap. 18, which contains the product of a squared matrix element and a shape function that accounts for the fact that the energies of the two levels are not infinitely sharp Sect. II D. For a mw magnetic field in the x direction, the relevant matrix element is $\langle +|\omega_{1}S_{x}|-\rangle$ . When the spread of the energy levels is identified with the EPR line shape, which we take to be a Lorentzian, one arrives at

4 $$v_{1}(\Omega )=\frac{1}{2}\omega_{1}^{2}\;\frac{R_{2S}}{R_{2S}^{2}+\Omega^{2}},$$

where $\Omega =\omega_{S}-\omega$ is the offset of the mw frequency ω from the electronic resonance frequency $\omega_{S}$ , and $R_{2S}$ is the electronic $T_{2}$ relaxation rate.

Formally, this perturbative result is valid only for short times chap. XIII. Its validity at long times, including the steady state, thus needs to be explicitly established pp. 30–32. In the next subsection, we show that Eq. ([Disp-formula Ch1.E4]) is consistent with the steady state of the Bloch equations.

**Figure 1 Ch1.F1:**
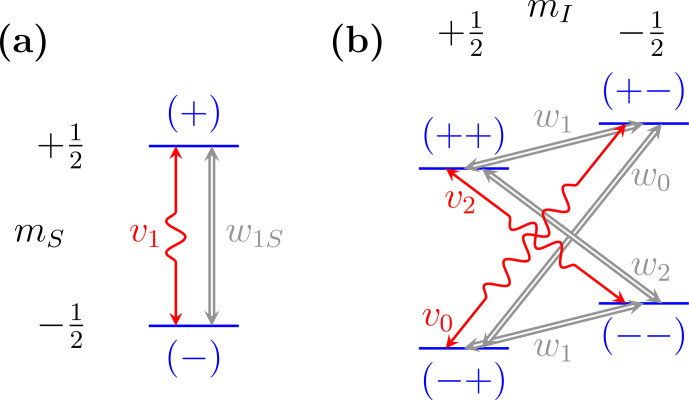
Energy levels of **(a)** a single electronic spin S=1/2 and **(b)** one electronic spin and one nuclear spin I=1/2 . Microwaves excite single-, zero- and double-quantum transitions (wiggly red arrows) with rate constants $v_{1}$ , $v_{0}$ and $v_{2}$ , respectively. Thermal relaxation (thick gray arrows) arises from coupling to external degrees of freedom.

### 2.2 Bloch equations

The effect of the microwaves on the two-level system in Fig. [Fig Ch1.F1]a is described exactly, and for all times, by the classical Bloch equations. The coherent part of these equations can be derived from the Liouville–von Neumann equation of the density matrix. Specifically, the evolution of the expectation value q=〈Q〉 of a general spin operator Q , under the action of a spin Hamiltonian H (in units of angular frequency), is

5 $$\dot{q}{|}_{\mathrm{coh}}=i\langle [H,Q]\rangle .$$

We describe the interaction of the electronic spins with the magnetic field using the following Hamiltonian in the rotating frame:

6 $$H=\Omega S_{z}+\omega_{1}S_{x}.$$

Here the first term accounts for the Zeeman interaction with the constant magnetic field $B_{0}$ (along the z axis) and the second for the interaction with the mw field $B_{1}$ (along x ).

Using Eq. ([Disp-formula Ch1.E6]) in Eq. ([Disp-formula Ch1.E5]), it is straightforward to obtain the coherent dynamics of $s_{z}=\langle S_{z}\rangle$ , $s_{y}=\langle S_{y}\rangle$ and $s_{x}=\langle S_{x}\rangle$ . After appending transverse and longitudinal relaxation by hand, one arrives at the familiar Bloch equations

7 $$\begin{array}{rl} {\dot{s}}_{x} & =-\Omega s_{y}-R_{2S}s_{x}\\ {\dot{s}}_{y} & =\Omega s_{x}-\omega_{1}s_{z}-R_{2S}s_{y}\\ {\dot{s}}_{z} & =\omega_{1}s_{y}-R_{1S}(s_{z}-s_{z}^{\mathrm{eq}}). \end{array}$$

Since the polarization $P_{S}$ corresponds to the expectation value of the spin operator $S_{z}$ , the rate equation Eq. ([Disp-formula Ch1.E2]) must be directly comparable to the third equation in Eq. ([Disp-formula Ch1.E7]). However, we see that the effect of the microwaves is modeled differently in the two equations. In the last Bloch equation, the microwaves couple $s_{z}$ to the transverse component $s_{y}$ . Such coupling is understandably missing in the rate equation, which describes the dynamics of $P_{S}$ without reference to the transverse components. Clearly, the two descriptions cannot be equivalent in general. Nevertheless, in spite of the fundamentally different ways the two descriptions model the microwaves, there is a regime where the Bloch equations and the rate equation are equivalent, not only approximately but exactly. This is the regime of steady state, as we show next.

At steady state, the transverse variables $s_{x,y}$ can be eliminated using the first two Bloch equations. From the first equation we find

8 $$s_{x}^{\mathrm{ss}}=-\frac{\Omega }{R_{2S}}s_{y}^{\mathrm{ss}},$$

where the superscript “ss” denotes steady state. Substituting this result into the second Bloch equation, we get

9 $$s_{y}^{\mathrm{ss}}=-\frac{\omega_{1}}{R_{2S}+\Omega \frac{1}{R_{2S}}\Omega }s_{z}^{\mathrm{ss}}.$$

We have thus expressed both transverse components in terms of the longitudinal component as follows:

10 $$s_{x,y}^{\mathrm{ss}}=\pm \left(\omega_{1}f_{x,y}\right)s_{z}^{\mathrm{ss}}$$

(the upper sign corresponds to x and the lower to y ), where we have defined the auxiliary functions

11 $$f_{y}=\frac{1}{R_{2S}+\Omega \frac{1}{R_{2S}}\Omega },\;f_{x}=\frac{\Omega }{R_{2S}}f_{y}.$$

Finally, substituting $s_{y}^{\mathrm{ss}}$ into the third Bloch equation in Eq. ([Disp-formula Ch1.E7]), we arrive at the following differential equation for $s_{z}$ at steady state:

12 $${\dot{s}}_{z}^{\mathrm{ss}}=-\omega_{1}^{2}f_{y}\;s_{z}^{\mathrm{ss}}-R_{1S}\left(s_{z}^{\mathrm{ss}}-s_{z}^{\mathrm{eq}}\right).$$

Although the time derivative on the left-hand side of Eq. ([Disp-formula Ch1.E12]) equals zero, the equation was written in this form to facilitate its comparison with the rate equation (Eq. [Disp-formula Ch1.E2]). Clearly, if the rate constant $v_{1}$ in Eq. ([Disp-formula Ch1.E2]) is selected such that

13 $$2v_{1}=\omega_{1}^{2}f_{y}=\omega_{1}^{2}\frac{1}{R_{2S}+\Omega \frac{1}{R_{2S}}\Omega },$$

then the steady state of $P_{S}$ will be identical to the steady state of $s_{z}$ . Incidentally, the $v_{1}$ in Eq. ([Disp-formula Ch1.E13]), which ensures that the two descriptions have the same steady state, is identical to the rate constant obtained from first-order perturbation theory (Eq. [Disp-formula Ch1.E4]). This will not be the case for the rate constants of the forbidden transitions, as we show in Sect. [Sec Ch1.S3].

Once the two descriptions are demonstrated to have identical steady states, the analysis of the Bloch equations can be terminated at this point since it will exactly follow the steady-state analysis of the rate equation. In the next section, where we determine the ZQ and DQ transition rates from the steady state of the spin dynamics, we will similarly need to consider only the evolution of $i_{z}=\langle I_{z}\rangle$ under the action of the microwaves. The balance between the mw irradiation and the nuclear $T_{1}$ relaxation will be handled on the level of the rate equation of the nuclear polarization.

For completeness, here we proceed one step further and solve Eq. ([Disp-formula Ch1.E12]) for $s_{z}^{\mathrm{ss}}$ recalling that the time derivative equals zero. The result is

14 $$s_{z}^{\mathrm{ss}}=\left(R_{1S}f_{z}\right)s_{z}^{\mathrm{eq}},$$

where we have defined

15 $$f_{z}=\frac{1}{R_{1S}+\omega_{1}^{2}f_{y}}.$$

The functions $f_{x}$ , $f_{y}$ and $f_{z}$ introduced in Eqs. ([Disp-formula Ch1.E11]) and ([Disp-formula Ch1.E15]) have units of time, and the factors enclosed in parentheses in Eqs. ([Disp-formula Ch1.E10]) and ([Disp-formula Ch1.E14]) are dimensionless. This information is collected in Table [Table Ch1.T1]. Since Eq. ([Disp-formula Ch1.E14]) is equivalent to Eq. ([Disp-formula Ch1.E3]), it provides an expression for the polarization factor p=1-s , where s is the familiar saturation factor of the (allowed) electronic transition.

**Table 1 Ch1.T1:** Functions characterizing the steady-state properties of the classical Bloch equations and the Bloch-like equations of the variables $g_{n}=\langle S_{n}I_{+}\rangle$ ( n=x,y,z ).

	Classical Bloch eqs.	Bloch-like eqs.
Unit of time	$f_{x},f_{y},f_{z}$	$F_{x},F_{y},F_{z}$
Dimensionless	$\omega_{1}f_{x},\omega_{1}f_{y},R_{1S}f_{z}$	$\omega_{1}F_{x},\omega_{1}F_{y},\delta F_{z}$

## 3 Forbidden transitions

The excitation of the allowed EPR transition considered above does not lead to simultaneous flips of the electronic and nuclear spins and is thus not capable of transferring polarization from the former to the latter. In contrast, the ZQ and DQ transitions involve simultaneous electron–nucleus spin flips (Fig. [Fig Ch1.F1]b) and drive the solid-state DNP effect. While these so-called forbidden transitions couple the nuclear and electronic polarizations, their influence on the latter is typically negligible compared to other mechanisms of electronic relaxation. It is therefore justified to write a rate equation for the electronic polarization considering only the allowed EPR transition, as we did in Sect. [Sec Ch1.S2]. The effect of the mw-induced ZQ and DQ transitions on the nuclear polarization is described in the current section.

### 3.1 Rate equation of the nuclear polarization

Let $n_{++}$ , $n_{+-}$ , $n_{-+}$ and $n_{--}$ be the populations of the levels of the four-level system in Fig. [Fig Ch1.F1]b. While their sum, $n=n_{++}+n_{+-}+n_{-+}+n_{--}$ , remains constant in time, the individual populations change due to the ZQ and DQ transitions with rate constants $v_{0}$ and $v_{2}$ as follows:

16 $$\begin{array}{rl} {\dot{n}}_{-+}{|}_{\mathrm{mw}} & =-{\dot{n}}_{+-}{|}_{\mathrm{mw}}=-v_{0}\left(n_{-+}-n_{+-}\right)\\ {\dot{n}}_{++}{|}_{\mathrm{mw}} & =-{\dot{n}}_{--}{|}_{\mathrm{mw}}=-v_{2}\left(n_{++}-n_{--}\right). \end{array}$$

It is implicitly assumed that $v_{0}\geq 0$ and $v_{2}\geq 0$ , as negative rate constants would not make physical sense.

The polarizations of the nuclear and electronic spins are

17 $$\begin{array}{rl} P_{I} & =\left[\left(n_{++}-n_{+-}\right)+\left(n_{-+}-n_{--}\right)\right]/n\\ P_{S} & =\left[\left(n_{++}-n_{-+}\right)+\left(n_{+-}-n_{--}\right)\right]/n. \end{array}$$

While, as before, $P_{S}^{\mathrm{eq}}<0$ , the sign of $P_{I}$ at thermal equilibrium will depend on the gyromagnetic ratio of the nuclear spin. We will assume protons; hence $\gamma_{I}>0$ and $P_{I}^{\mathrm{eq}}>0$ . Differentiating the definition of $P_{I}$ in Eq. ([Disp-formula Ch1.E17]) with respect to time, and using Eq. ([Disp-formula Ch1.E16]), we obtain

18 $$\begin{array}{rl} {\dot{P}}_{I}{|}_{\mathrm{mw}} & =-v_{0}\left(P_{I}-P_{S}\right)-v_{2}\left(P_{I}+P_{S}\right)\\ & =-\left(v_{2}+v_{0}\right)P_{I}-\left(v_{2}-v_{0}\right)P_{S}\\ & =-v_{+}P_{I}-v_{-}P_{S}, \end{array}$$

which shows that mw excitation of the forbidden transitions couples the evolution of the nuclear polarization to the polarization of the electrons. This coupling is responsible for the solid effect. Because one always encounters either the difference or the sum of $v_{0}$ and $v_{2}$ , in the third equality of Eq. ([Disp-formula Ch1.E18]) we introduced

19 $$v_{\pm }=v_{2}\pm v_{0}.$$

In fact, as we show later, the individual rates $v_{0}$ and $v_{2}$ may become negative and thus meaningless from the rate-equation point of view.

Although in the current paper we are only interested in the rates that describe the effect of the microwaves (i.e., the red arrows in Fig. [Fig Ch1.F1]), we also discuss thermal relaxation as it is essential for reaching steady state.

Thermal relaxation of the nuclear spins due to their coupling to the electronic spins acts analogously to Eq. ([Disp-formula Ch1.E18]) after replacing the rates $v_{0,2}$ by $w_{0,2}$ and the polarizations by their deviations from thermal equilibrium. Further including nuclear $T_{1}$ relaxation due to mechanisms other than the coupling to the electrons, we arrive at

20 $$\begin{array}{rl} {\dot{P}}_{I}{|}_{\mathrm{th}} & =-R_{1I}^{0}\left(P_{I}-P_{I}^{\mathrm{eq}}\right)-2w_{1}\left(P_{I}-P_{I}^{\mathrm{eq}}\right)\\ & \;-w_{+}\left(P_{I}-P_{I}^{\mathrm{eq}}\right)-w_{-}\left(P_{S}-P_{S}^{\mathrm{eq}}\right), \end{array}$$

where $R_{1I}^{0}$ is the nuclear $T_{1}$ relaxation rate in the absence of the polarizing agent, and, analogously to Eq. ([Disp-formula Ch1.E19]),

21 $$w_{\pm }=w_{2}\pm w_{0}.$$

The cross-relaxation rate $w_{-}$ is seen to couple the dynamics of $P_{I}$ to $P_{S}$ . This coupling leads to the Overhauser effect.

From Eq. ([Disp-formula Ch1.E20]), the total nuclear $T_{1}$ relaxation rate (i.e., in the presence of the free radical) is identified as $R_{1I}=R_{1I}^{0}+2w_{1}+w_{+}$ . Combining the contributions of mw excitation (Eq. [Disp-formula Ch1.E18]) and relaxation (Eq. [Disp-formula Ch1.E20]), we arrive at the following rate equation for the nuclear polarization:

22 $$\begin{array}{rl} {\dot{P}}_{I} & =-R_{1I}\left(P_{I}-P_{I}^{\mathrm{eq}}\right)-w_{-}\left(P_{S}-P_{S}^{\mathrm{eq}}\right)\\ & \;-v_{+}P_{I}-v_{-}P_{S}. \end{array}$$

As the rate equations are only used in our analysis to describe the steady state, we solve Eq. ([Disp-formula Ch1.E22]) at steady state and express the nuclear polarization under cw irradiation in terms of the equilibrium polarizations:

23 $$P_{I}^{\mathrm{ss}}=\frac{R_{1I}}{R_{1I}+v_{+}}P_{I}^{\mathrm{eq}}+\frac{sw_{-}}{R_{1I}+v_{+}}P_{S}^{\mathrm{eq}}-\frac{pv_{-}}{R_{1I}+v_{+}}P_{S}^{\mathrm{eq}}.$$

(We used Eq. [Disp-formula Ch1.E3] for the steady-state electronic polarization, and s=1-p .)

DNP is generally quantified through the enhancement of the nuclear polarization,

24 $$\epsilon =P_{I}^{\mathrm{ss}}/P_{I}^{\mathrm{eq}}-1,$$

which is defined such that it equals zero at thermal equilibrium. Taking into account that $P_{S}^{\mathrm{eq}}/P_{I}^{\mathrm{eq}}=-|\gamma_{S}|/\gamma_{I}$ , where $\gamma_{S}$ and $\gamma_{I}$ are the gyromagnetic ratios of the electronic and nuclear spins, from Eq. ([Disp-formula Ch1.E23]) we obtain

25 $$\epsilon =\epsilon_{\mathrm{SE}}+\epsilon_{\mathrm{OE}}+\left(p_{X}-1\right)$$

with

26 $$\begin{array}{rl} \epsilon_{\mathrm{SE}} & =\frac{pv_{-}}{R_{1I}+v_{+}}\frac{\left|\gamma_{S}\right|}{\gamma_{I}},\;\epsilon_{\mathrm{OE}}=-\frac{sw_{-}}{R_{1I}+v_{+}}\frac{\left|\gamma_{S}\right|}{\gamma_{I}}\\ p_{X} & =\frac{R_{1I}}{R_{1I}+v_{+}}. \end{array}$$

The first two additive contributions to the DNP enhancement correspond to, respectively, the solid and Overhauser effects. The last one is due to neither of them. Since it does not scale with the ratio of the gyromagnetic ratios, it should be negligible in all cases of practical interest. Note that $p_{X}$ is similar to the electronic polarization factor p in Eq. ([Disp-formula Ch1.E3]) but with $R_{1S}$ and $2v_{1}$ replaced by $R_{1I}$ and $v_{0}+v_{2}$ , respectively.

For the expressions in Eq. ([Disp-formula Ch1.E26]) to have a predictive value, it is necessary to express the rates $v_{\pm }$ in terms of more fundamental quantities. This is done using first-order perturbation theory, under the assumption that the dipolar interaction between the electronic and nuclear spins is much smaller than the nuclear splitting [Bibr bib1.bibx1]. Because the dipolar interaction mixes the Zeeman energy levels depicted in Fig. [Fig Ch1.F1]b, the ZQ and DQ transitions become weakly allowed. To first order, the mixed states are of the form (--)+q(-+) [Bibr bib1.bibx1], with mixing parameter

27 $$q=\frac{1}{4}\frac{D_{\mathrm{dip}}}{\omega_{I}}\frac{-3\mathrm{cos}\theta \mathrm{sin}\theta \;{\text{e}}^{i\phi }}{r^{3}}.$$

Here, $D_{\mathrm{dip}}=(\mu_{0}/4\pi )\hbar \gamma_{S}\gamma_{I}$ is the dipolar constant and ( r,θ,ϕ ) are the spherical polar coordinates of the relative position vector of the spins. The probability amplitude of the microwaves to excite a transition between the mixed energy levels is then proportional to $\omega_{1}q$ . Combining the probability of excitation with the Lorentzian spread of the electronic energy levels, one arrives at the rate constants [Bibr bib1.bibx34]

28 $$v_{0,2}(\Omega )=4\left(q^{*}q\right)\;v_{1}\left(\Omega \pm \omega_{I}\right),$$

where $v_{1}$ is the rate of the allowed (single-quantum) EPR transition (Eq. [Disp-formula Ch1.E4]). In essence, the rates of the ZQ and DQ transitions are obtained by shifting the rate of the allowed transition along the frequency axis by $\pm \omega_{I}$ and reducing its magnitude through multiplication by $4|q{|}^{2}$ .

We observe that in this approach the rates of the forbidden transitions acquire a factor of $\omega_{I}^{-2}$ from $|q{|}^{2}$ and a factor of $\omega_{1}^{2}$ from the mw excitation (Eq. [Disp-formula Ch1.E4]), without any room for non-trivial cross talk between these two frequencies. Such cross talk is also not provided by the Lorentzian dependence on Ω . Similar to Eq. ([Disp-formula Ch1.E28]), the rate constants that we will obtain in the next subsection will also contain $\omega_{1}^{2}$ and $D_{\mathrm{dip}}^{2}$ as multiplicative factors. However, their offset dependence will couple $\omega_{1}$ and $\omega_{I}$ in a non-trivial way, which will reduce to the classical expression when $\omega_{1}\ll \omega_{I}$ but will predict qualitatively different dependence when $\omega_{1}$ is similar to or larger than $\omega_{I}$ (Sect. [Sec Ch1.S6.SS1]).

### 3.2 Generalized Bloch equations for the solid effect

In this section, we obtain alternative expressions for the forbidden-transition rates $v_{\pm }$ from the steady state of the exact quantum dynamics. We start by deriving equations of motion for the expectation values of the operators relevant to the solid effect. To use Eq. ([Disp-formula Ch1.E5]), we need to first specify the Hamiltonian guiding the dynamics.

We will consider the minimal solid-effect spin Hamiltonian [Bibr bib1.bibx33]

29 $$H=\Omega S_{z}+\omega_{1}S_{x}-\omega_{I}I_{z}+\frac{1}{2}\left(A_{1}^{*}S_{z}I_{+}+A_{1}S_{z}I_{-}\right),$$

which is in the rotating frame for the electronic spin and in the laboratory frame for the nuclear spin. The first two terms are the same as in Eq. ([Disp-formula Ch1.E6]). The third term describes the nuclear Zeeman interaction. The sign of $\omega_{I}$ is negative since we assumed a nuclear spin with positive gyromagnetic ratio. The last two terms in Eq. ([Disp-formula Ch1.E29]) account for the dipolar interaction between the electronic and nuclear spins. We have truncated this interaction by dropping all non-secular terms containing $S_{x}$ and $S_{y}$ . Similar to the assumption behind the derivation of the mixing factor (Eq. [Disp-formula Ch1.E27]), we take the dipolar interaction to be small compared to the nuclear Zeeman splitting and drop the secular term proportional to $S_{z}I_{z}$ . The remaining, pseudosecular terms scale with the dipolar coupling [Bibr bib1.bibx33]

30 $$A_{1}=D_{\mathrm{dip}}\frac{-3\mathrm{cos}\theta \mathrm{sin}\theta }{r^{3}}{\text{e}}^{i\phi },$$

where $D_{\mathrm{dip}}/2\pi \approx 79.066\;\mathrm{kHz}\;{\mathrm{nm}}^{3}$ for protons. The subscript of $A_{1}$ indicates that its angular dependence is identical to the second-degree spherical harmonic of order m=1 .

We start our derivation of equations of motion with $i_{z}=\langle I_{z}\rangle$ , as it corresponds to the nuclear polarization. There is no contribution from the first three terms in the Hamiltonian (Eq. [Disp-formula Ch1.E29]) as $I_{z}$ commutes with all of them (Eq. [Disp-formula Ch1.E5]). From the commutator with the dipolar terms we obtain

31 $${\dot{i}}_{z}{|}_{\mathrm{coh}}=i\frac{1}{2}\left(A_{1}g_{z}^{*}-A_{1}^{*}g_{z}\right)=-\text{Re}\{iA_{1}^{*}g_{z}\},$$

where

32 $$g_{n}=\langle S_{n}I_{+}\rangle \;(n=x,y,z).$$

Proceeding in the same way, we first find

33 $${\dot{g}}_{z}{|}_{\mathrm{coh}}=-i\omega_{I}g_{z}+\omega_{1}g_{y}-i\left(A_{1}/4\right)i_{z}$$

and then

34 $$\begin{array}{rl} {\dot{g}}_{y}{|}_{\mathrm{coh}} & =\Omega g_{x}-i\omega_{I}g_{y}-\omega_{1}g_{z}+\left(A_{1}/4\right)s_{x}\\ {\dot{g}}_{x}{|}_{\mathrm{coh}} & =-i\omega_{I}g_{x}-\Omega g_{y}-\left(A_{1}/4\right)s_{y}. \end{array}$$

The chain of dynamical equations can be terminated at this stage, as $s_{x,y}$ obey the classical Bloch equations discussed above. (The dynamics of the electronic spin was taken to be independent of its dipolar coupling with the nuclei.)

In addition to the coherent evolution considered so far, $g_{z}=\langle S_{z}I_{+}\rangle$ and $g_{x,y}=\langle S_{x,y}I_{+}\rangle$ are expected to decay with rates $R_{1S}+R_{2I}$ and $R_{2S}+R_{2I}$ , respectively. Neglecting $R_{2I}$ compared to $R_{1S}$ and $R_{2S}$ , we arrive at the following system of coupled differential equations:

35 $$\begin{array}{rl} {\dot{g}}_{x} & =-\left(R_{2S}+i\omega_{I}\right)g_{x}-\Omega g_{y}-\left(A_{1}/4\right)s_{y}\\ {\dot{g}}_{y} & =\Omega g_{x}-\left(R_{2S}+i\omega_{I}\right)g_{y}-\omega_{1}g_{z}+\left(A_{1}/4\right)s_{x}\\ {\dot{g}}_{z} & =-\left(R_{1S}+i\omega_{I}\right)g_{z}+\omega_{1}g_{y}-i\left(A_{1}/4\right)i_{z}. \end{array}$$

Equations ([Disp-formula Ch1.E31]) and ([Disp-formula Ch1.E35]), supplemented by the Bloch equations (Eq. [Disp-formula Ch1.E7]), constitute the generalization of the Bloch equations to the four-level system in Fig. [Fig Ch1.F1]b as relevant to the solid effect. If desired, one can also supplement Eq. ([Disp-formula Ch1.E31]) with nuclear $T_{1}$ relaxation. However, because our aim is to identify the rates $v_{\pm }$ , this is not necessary. In any case, the balance between thermal relaxation and mw excitation at steady state was already analyzed using the rate-equation formalism (Sect. [Sec Ch1.S3.SS1]).

Analogously to our treatment of the Bloch equations (Sect. [Sec Ch1.S2.SS2]), we will now use the condition of steady state to eliminate all variables except the polarizations $i_{z}$ and $s_{z}$ . From the steady state of the first equation in Eq. ([Disp-formula Ch1.E35]) we get

36 $$g_{x}^{\mathrm{ss}}=-\frac{\Omega }{R_{2S}+i\omega_{I}}g_{y}^{\mathrm{ss}}-\frac{A_{1}/4}{R_{2S}+i\omega_{I}}s_{y}^{\mathrm{ss}}.$$

Substituting into the second equation of Eq. ([Disp-formula Ch1.E35]) we find

37 $$g_{y}^{\mathrm{ss}}=-\omega_{1}F_{y}\;g_{z}^{\mathrm{ss}}+\left(A_{1}/4\right)\left(F_{y}\;s_{x}^{\mathrm{ss}}-F_{x}\;s_{y}^{\mathrm{ss}}\right),$$

where we introduced the complex-valued functions

38 $$\begin{array}{rl} F_{y} & =\frac{1}{R_{2S}+i\omega_{I}+\Omega \frac{1}{R_{2S}+i\omega_{I}}\Omega }\\ F_{x} & =\frac{\Omega }{R_{2S}+i\omega_{I}}F_{y}, \end{array}$$

which generalize the functions in Eq. ([Disp-formula Ch1.E11]) by supplementing their relaxation rates with an imaginary part. Like their real analogs, $F_{x,y}$ have units of time (Table [Table Ch1.T1]).

Substituting $g_{y}^{\mathrm{ss}}$ into the last equation of Eq. ([Disp-formula Ch1.E35]) and solving for $g_{z}$ at steady, we find

39 $$g_{z}^{\mathrm{ss}}=-i\left(A_{1}/4\right)F_{z}i_{z}^{\mathrm{ss}}+\left(A_{1}/4\right)F_{z}\left(\omega_{1}F_{y}\;s_{x}^{\mathrm{ss}}-\omega_{1}F_{x}\;s_{y}^{\mathrm{ss}}\right),$$

where the function

40 $$F_{z}=\frac{1}{R_{1S}+i\omega_{I}+\omega_{1}^{2}F_{y}}$$

generalizes Eq. ([Disp-formula Ch1.E15]) of the classical Bloch equations. Finally, we substitute $g_{z}^{\mathrm{ss}}$ into the equation of $i_{z}$ (Eq. [Disp-formula Ch1.E31]). Factoring out the dipolar coupling as

41 $$\delta^{2}=\left(A_{1}^{*}A_{1}\right)/4,$$

at steady state, Eq. ([Disp-formula Ch1.E31]) becomes

42 $$\begin{array}{rl} {\dot{i}}_{z}^{\mathrm{ss}}{|}_{\mathrm{coh}}= & -\delta^{2}\text{Re}\{F_{z}\}i_{z}^{\mathrm{ss}}-\delta^{2}\text{Re}\{iF_{z}\left(\omega_{1}F_{y}\right)\}s_{x}^{\mathrm{ss}}\\ & -\delta^{2}\text{Re}\{iF_{z}\left(-\omega_{1}F_{x}\right)\}s_{y}^{\mathrm{ss}}. \end{array}$$

We have thus managed to eliminate the three electron–nucleus coherences $g_{n}$ .

To further eliminate the electronic coherences from Eq. ([Disp-formula Ch1.E42]), we recall that at steady state the transverse components $s_{x,y}$ are algebraically related to $s_{z}$ (Eq. [Disp-formula Ch1.E10]). Hence,

43 $${\dot{i}}_{z}^{\mathrm{ss}}{|}_{\mathrm{coh}}=-\delta^{2}\text{Re}\{F_{z}\}i_{z}^{\mathrm{ss}}-\delta^{2}\omega_{1}^{2}\text{Re}\{iF_{z}\left(F_{y}\;f_{x}+F_{x}\;f_{y}\right)\}s_{z}^{\mathrm{ss}}.$$

As the right-hand side of Eq. ([Disp-formula Ch1.E43]) contains only $i_{z}$ and $s_{z}$ , it can be directly compared with the rate equation that accounts for the contribution of the microwaves to the time derivative of $P_{I}$ (Eq. [Disp-formula Ch1.E18]). The comparison allows us to identify the two phenomenological rate constants of the forbidden transitions as

44 $$v_{+}=\delta^{2}\text{Re}\{F_{z}\},\;v_{-}=\delta^{2}\omega_{1}^{2}\text{Re}\{iF_{z}\left(F_{y}\;f_{x}+F_{x}\;f_{y}\right)\}.$$

When used with these two rate constants, the rate equation of $P_{I}$ is guaranteed to have the correct steady state.

The above non-perturbative derivation of the rate constants $v_{\pm }$ is the main analytical contribution of the current paper. In Sect. [Sec Ch1.S6], we will explore the predictions of these expressions, as well as their relationship to the classical perturbative rates (Eq. [Disp-formula Ch1.E28]). Before that, in the next section, we revisit the equations of motion (Eqs. [Disp-formula Ch1.E31] and [Disp-formula Ch1.E35]) and the Bloch equations (Eq. [Disp-formula Ch1.E7]), which constitute a system of seven coupled differential equations. The steady state of this system of equations is examined in Sect. [Sec Ch1.S5].

## 4 Making sense of the spin dynamics

The Bloch equations (Eq. [Disp-formula Ch1.E7]) are coupled differential equations describing the time evolution of three dynamical variables. When the number of coupled equations is small, it is possible to form a mental picture of the dynamical interconnections between the variables by examining the written equations. In the case of more than three variables, however, gaining insight into the dynamics by simply looking at the written equations becomes harder.

The need to make sense of several coupled differential equations also arises in the context of chemical reaction kinetics, where the concentrations of the reactants change in time. When the number of chemical species is small, it is sufficient to write down the kinetic equations for the concentrations. However, when one deals with the reactions of even a relatively simple metabolic pathway, like glycolysis or the citric acid cycle, the rate equations are almost never written down explicitly. Instead, they are represented in a visual way by drawing arrows between the names of the chemical species that are interconverted by the reactions.

Following the same logic, we represent the dynamical variables $s_{x}$ , $s_{y}$ and $s_{z}$ of the classical Bloch equations (Eq. [Disp-formula Ch1.E7]) as nodes and the various interactions that couple their dynamics as arrows (Fig. [Fig Ch1.F2]a). The time derivative of each variable is calculated by summing the contributions of all arrows that point *into* its node, where the contribution of an arrow is obtained by multiplying the weight of the arrow by the variable from which it *originates*. Differently from the representation of chemical reactions, here an arrow does not deplete the node at its origin but only contributes to the node at its pointed end. In addition, as our arrows do not have the physical interpretation of reaction rate constants, their weights may also be negative.

The two orange arrows in Fig. [Fig Ch1.F2]a, which flow into the node of $s_{x}$ , correspond to the two terms on the right-hand side of the first Bloch equation in Eq. ([Disp-formula Ch1.E7]). The arrow with weight -Ω originates from $s_{y}$ and thus contributes $-\Omega s_{y}$ to the time derivative of $s_{x}$ . The other orange arrow originates from $s_{x}$ and accounts for the decay of this variable with the rate constant $R_{2S}$ of the transverse relaxation. We refer to such arrows that leave a node and enter the same node as self-arrows. To prevent positive feedback and thus ensure dynamical stability, the total contribution of self-arrows (in case several such arrows point into a node) should be positive. We will generally write the weight of a self-arrow with an explicit negative sign, which we place inside the loop formed by the arrow.

Similarly, the three blue arrows in Fig. [Fig Ch1.F2]a, which flow into the node of $s_{y}$ , correspond to the three terms on the right-hand side of the second Bloch equation in Eq. ([Disp-formula Ch1.E7]). The remaining three arrows, which flow into $s_{z}$ , correspond to the right-hand side of the last Bloch equation. Rather then using the same color for these three arrows, we have indicated the contribution of mw irradiation with red and the contribution of relaxation with gray, in line with the colors used in Fig. [Fig Ch1.F1]a. In any case, the colors of the arrows do not play a role in the correspondence between the differential equation and its visual representation. Because the equilibrium value $s_{z}^{\mathrm{eq}}$ is a constant parameter in the Bloch equations, there are no arrows flowing into its node. A node is shaded gray when the corresponding variable remains constant in time.

While Fig. [Fig Ch1.F2]a contains exactly the same information as the Bloch equations (Eq. [Disp-formula Ch1.E7]), all dynamical interconnections between the variables are now visually accessible. For example, the loop formed by the two arrows with weights $-\omega_{1}$ and $\omega_{1}$ between the variables $s_{y}$ and $s_{z}$ corresponds to rotation in the y – z plane with angular velocity equal to $\omega_{1}$ . In other words, this loop is a visual manifestation of the Rabi nutation driven by the microwaves. There is a similar loop between the variables $s_{x}$ and $s_{y}$ , which corresponds to rotation with angular velocity Ω in the x – y plane. This is the Larmor precession, as seen in the rotating frame. Since all other arrows correspond to relaxation, the diagram in Fig. [Fig Ch1.F2]a confirms in a visual way that the coherent part of the Bloch equations consists of two rotations.

**Figure 2 Ch1.F2:**
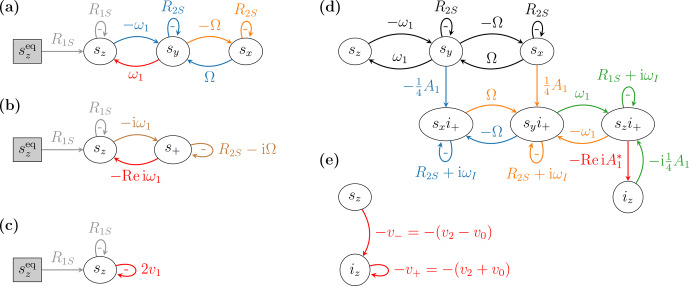
**(a)** Real-valued and **(b)** complex-valued classical Bloch equations and **(c)** corresponding dynamics according to the rate equation of the electronic polarization. **(d)** Spin dynamics of relevance to the solid effect and **(e)** corresponding dynamics implied by the rate equation of the nuclear polarization.

At this point we mention that instead of working with the real-valued Bloch equations (Eq. [Disp-formula Ch1.E7]), one could form the dynamical variable $s_{+}=s_{x}+is_{y}$ and work with the complex-valued Bloch equations

45 $$\begin{array}{rl} {\dot{s}}_{+} & =-\left(R_{2S}-i\Omega \right)s_{+}-i\omega_{1}s_{z}\\ {\dot{s}}_{z} & =-R_{1S}\left(s_{z}-s_{z}^{\mathrm{eq}}\right)-\text{Re}\{i\omega_{1}s_{+}\}. \end{array}$$

These two differential equations are depicted in Fig. [Fig Ch1.F2]b. Notably, the rotation in the x – y plane with angular velocity Ω (i.e., the Larmor precession) has now become the imaginary part of the self-arrow of $s_{+}$ , whose real part is the $T_{2}$ relaxation rate.

In Eq. ([Disp-formula Ch1.E45]) we arbitrarily retained $s_{+}$ and dropped $s_{-}$ , thus reducing the number of variables in the diagrammatic representation from three to two. (The analogous reduction will be more substantial in the case of the coupled electron–nucleus system.) Note, however, that the contribution of $s_{-}$ is recovered when the real part of $is_{+}$ is evaluated to calculate the time derivative of $s_{z}$ in the second line of Eq. ([Disp-formula Ch1.E45]).

In Fig. [Fig Ch1.F2]c we have represented the dynamics of $s_{z}$ which is implied by the rate equation of the electronic polarization (Eq. [Disp-formula Ch1.E2]). The visual comparison of this dynamics with the Bloch equations above it makes clear that the rate $v_{1}$ of the allowed EPR transition is supposed to account in some effective way for the coupling between $s_{z}$ and $s_{y}$ (due to $\omega_{1}$ ) and for the dynamics of the transverse components (due to Ω and $R_{2S}$ ). Indeed, the rate constant $v_{1}$ in Eq. ([Disp-formula Ch1.E13]) is a function of $\omega_{1}$ , Ω and $R_{2S}$ .

In Fig. [Fig Ch1.F2]d we show the system of seven coupled differential equations that play a role in the solid effect (Eqs. [Disp-formula Ch1.E31] and [Disp-formula Ch1.E35] and the Bloch equations). For clarity, the nodes $g_{n}$ ( n=x,y,z ) are labeled as $s_{n}i_{+}$ in the figure. Black arrows correspond to the classical Bloch equations. Blue, orange and green arrows, which flow into the nodes $g_{x}$ , $g_{y}$ and $g_{z}$ , respectively, correspond to the right-hand sides of the three equations in Eq. ([Disp-formula Ch1.E35]). The red arrow flowing into the node of $i_{z}$ corresponds to the right-hand side of Eq. ([Disp-formula Ch1.E31]). Note that the weight of the red arrow involves taking a real part, just like in the complex-valued Bloch equations. Thus, although we only show the dynamics of the coherences $S_{n}I_{+}$ , at this point the effect of the coherences $S_{n}I_{-}$ is also included. In other words, if we did not take the real part, we would need to represent 10 coupled differential equations, rather than 7.

The graphical representation of the spin dynamics in Fig. [Fig Ch1.F2]d lays bare the overall topology of the dynamical connections between the seven variables. For example, note that the Bloch-equation pattern connecting the top three nodes (black arrows) is recapitulated between the nodes of the coherences $g_{n}$ below them. Indeed, between the electron–nucleus coherences one recognizes the loops that correspond to Rabi nutation and Larmor precession. Due to the involvement of the nuclear spin operator $I_{+}$ , this second set of Bloch equations is “shifted” by the nuclear Larmor frequency, as evidenced by the imaginary part of the self-arrows of $g_{n}$ . The link between the electronic Bloch equations and these new Bloch equations that describe the dynamics of the S – I coherences is established by the dipolar coupling ( $A_{1}$ ), which connects the two sets of Bloch equations such that the y variable of one of them feeds into the x variable of the other and vice versa. The same dipolar interaction also connects $g_{z}$ to the nuclear polarization through the red arrow in Fig. [Fig Ch1.F2]d. Although the coherences $S_{n}I_{-}$ are not explicitly modeled, their contribution is recovered when we feed a real value into the time derivative of $i_{z}$ , as discussed above. At this stage, Bloch-like equations shifted by $+\omega_{I}$ (shown) and by $-\omega_{I}$ (not shown) contribute symmetrically to the nuclear polarization.

All interactions in the Hamiltonian (Eq. [Disp-formula Ch1.E29]) lead to rotations, which are manifested as loops between two variables formed by arrows with opposite weights. Although such loops are also formed between the variables $s_{y}$ and $g_{x}$ , and between $s_{x}$ and $g_{y}$ , we have not shown the arrows that originate at $g_{x}$ and $g_{y}$ and flow into, respectively, $s_{y}$ and $s_{x}$ . These arrows, which would complete the loops of the dipolar interaction, are dropped because their contribution to the electronic dynamics is neglected.

For comparison, in Fig. [Fig Ch1.F2]e we recall the description of the same spin dynamics according to the rate-equation formalism (Eq. [Disp-formula Ch1.E18]). Clearly, the two rates $v_{\pm }$ should summarize in some faithful way the complexity of the proper, quantum-mechanical dynamics in Fig. [Fig Ch1.F2]d. In particular, the rate $v_{-}$ should account for the pathways from $s_{z}$ to $i_{z}$ and the rate $v_{+}$ for the pathways from $i_{z}$ into the coherences $g_{n}$ and back to $i_{z}$ .

By examining the pathways from $s_{z}$ to $i_{z}$ we gain visual understanding of the mechanism of dynamical coupling between the electronic and nuclear polarizations in the solid effect. The two possible paths for reaching $i_{z}$ from $s_{z}$ following the “flow” of the arrows are shown in Fig. [Fig Ch1.F3]a. Both paths consist of four steps, not counting the mixing of the transverse components by ±Ω . First, the mw excitation ( $\omega_{1}$ ) generates the transverse components $s_{y,x}$ from $s_{z}$ . This step is described by the classical Bloch equations. Then, the dipolar coupling ( $A_{1}$ ) generates the coherences $g_{x,y}$ from $s_{y,x}$ . These are then converted to $g_{z}$ by the mw excitation, and finally the dipolar interaction transforms $g_{z}$ to $i_{z}$ . Observe that the weights $\omega_{1}$ and $A_{1}$ appear twice along each path; thus both paths scale as $\omega_{1}^{2}|A_{1}{|}^{2}$ . Since these paths contribute to the rate constant $v_{-}$ , it should also scale with the mw power and the square of the dipolar interaction, which is in agreement with the perturbative rates in Eq. ([Disp-formula Ch1.E28]).

In addition to the weights considered above, both paths in Fig. [Fig Ch1.F3]a also traverse arrows with weights ±Ω . Thus, on resonance (i.e., Ω=0 ), the possibility of polarization transfer is severed. This observation does not appear to be particularly useful as the forbidden transitions are driven at $\Omega \approx \pm \omega_{I}$ anyway. However, since going along an arrow with weight ±Ω amounts to multiplication by Ω , we realize that crossing from the left side of the dynamical network to the right side involves change of parity in Ω . In other words, because $s_{z}$ is an even function of the frequency offset, its effect on $i_{z}$ must be odd in Ω . This is the reason for the anti-symmetric field profile of the solid effect (in contrast to the symmetric profile of the Overhauser effect). The diagram makes clear that the solid effect is odd in Ω for the same reason that $s_{x}$ is odd. This point is further examined in Sect. [Sec Ch1.S5].

**Figure 3 Ch1.F3:**
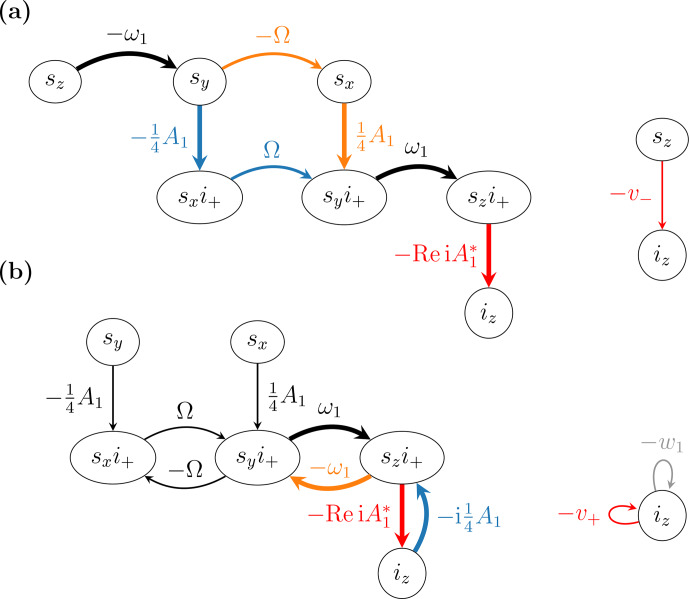
Pathways **(a)** from $s_{z}$ to $i_{z}$ contributing to the rate $v_{-}$ and **(b)** from $i_{z}$ to $i_{z}$ contributing to the rate $v_{+}$ .

In Fig. [Fig Ch1.F3]b we have highlighted the arrows that contribute to the self-loop of $i_{z}$ with weight $v_{+}$ . Again there are two different possible paths: one consists of two steps and the other of four. The shorter path from $i_{z}$ to $g_{z}$ (blue arrow) and back to $i_{z}$ (red arrow) relies only on the dipolar interaction between the electronic and nuclear spins and must be active even in the absence of mw excitation. The longer path additionally goes from $g_{z}$ to $g_{x,y}$ (the latter are mixed by Ω ) and back and contributes only under mw irradiation. Considering that thermal relaxation and mw excitation are treated separately, we realize that the short loop in fact contributes to the nuclear $T_{1}$ relaxation (more precisely to the rate $w_{1}$ in Fig. [Fig Ch1.F1]b); hence its contribution should be removed when calculating the rate $v_{+}$ .

On the basis of this observation, we now modify the analytical expression for $v_{+}$ that we gave in Eq. ([Disp-formula Ch1.E44]). Since the nuclear $T_{1}$ is typically measured with the microwaves switched off, we identify the $\omega_{1}$ -independent part of $F_{z}$ (Eq. [Disp-formula Ch1.E40]), namely

46 $$F_{z}\left(\omega_{1}=0\right)={\left(R_{1S}+i\omega_{I}\right)}^{-1},$$

as contributing to relaxation. The corrected form of the first equality in Eq. ([Disp-formula Ch1.E44]) is thus

47 $$v_{+}=\delta^{2}\text{Re}\{F_{z}-{\left(R_{1S}+i\omega_{I}\right)}^{-1}\}.$$

Having a visual representation of the spin dynamics was thus helpful to identify an aspect that would be harder to identify on the level of the written equations.

## 5 Analyzing the steady state

The diagrammatic representations of the previous section showed that the quantum-mechanical dynamics consists of several simultaneous rotations that mix the expectation values of the various spin operators. In spite of the complicated time evolution that such interconnected rotations may generally lead to, relatively simple algebraic relationships between the variables emerged at steady state (Sects. [Sec Ch1.S2.SS2] and [Sec Ch1.S3.SS2]).

The steady-state relationships of the Bloch equations, which were given in Eqs. ([Disp-formula Ch1.E10]) and ([Disp-formula Ch1.E14]), are depicted diagrammatically in Fig. [Fig Ch1.F4]a. Because we deal with algebraic (as opposed to differential) equations, the inflowing arrows now contribute directly to the value of the variable inside the node and not to its time derivative. To make this distinction visually clear, we use a rectangular node when the variable itself is obtained by adding the contributions of all inflowing arrows. In addition, we use dashed arrows to signal that the mathematical relationships hold only at steady state. In contrast, the solid arrows of the previous section represented fundamental, causal relationships between the variables governing their dynamics at all times.

**Figure 4 Ch1.F4:**
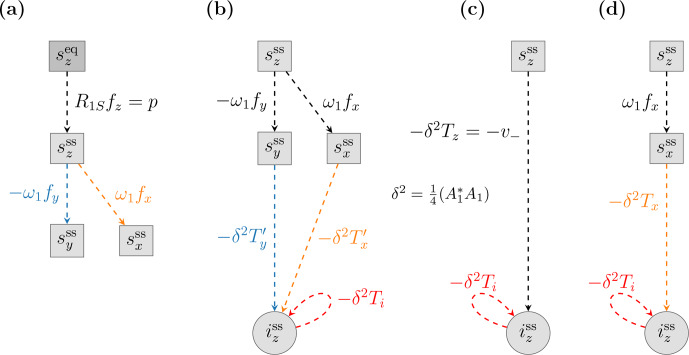
Algebraic relations between the dynamical variables at steady state. **(a)** Transfer functions of the Bloch equations. **(b, c, d)** Transfer functions describing the steady-state relationship between the time derivative of $i_{z}$ (output) and different choices of the electronic input.

It is convenient to think of the steady-state Bloch equations as a system that takes the Boltzmann polarization $s_{z}^{\mathrm{eq}}$ as an input and produces the outputs $s_{x,y}^{\mathrm{ss}}$ , as suggested graphically in Fig. [Fig Ch1.F4]a. Each dashed arrow can thus be viewed as a transfer function that multiplies the variable at its input to produce the variable at its output. The weights of the arrows in Fig. [Fig Ch1.F4]a are dimensionless (Table [Table Ch1.T1]).

Equation ([Disp-formula Ch1.E43]), from which we identified the rates $v_{\pm }$ , is depicted in Fig. [Fig Ch1.F4]b and, equivalently, in Fig. [Fig Ch1.F4]c. The three colored arrows in Fig. [Fig Ch1.F4]b correspond to the three terms on the right-hand side of Eq. ([Disp-formula Ch1.E42]), before the transverse components $s_{x,y}^{\mathrm{ss}}$ were replaced by $s_{z}^{\mathrm{ss}}$ . Specifically,

48 $$\begin{array}{rl} & T_{i}=\text{Re}\{F_{z}\},\;T_{x}^{\prime }=\text{Re}\{iF_{z}\left(\omega_{1}F_{y}\right)\},\\ & T_{y}^{\prime }=\text{Re}\{iF_{z}\left(-\omega_{1}F_{x}\right)\}. \end{array}$$

The cumulative transfer function from $s_{z}$ to $i_{z}$ (Fig. [Fig Ch1.F4]c) is obtained by adding the contributions of the two parallel paths in Fig. [Fig Ch1.F4]b. The sum

49 $$\begin{array}{rl} T_{z} & =\omega_{1}f_{x}T_{x}^{\prime }-\omega_{1}f_{y}T_{y}^{\prime }\\ & =\omega_{1}^{2}\;\text{Re}\{iF_{z}\left(F_{y}f_{x}+F_{x}f_{y}\right)\}=v_{-}/\delta^{2} \end{array}$$

was already evaluated in Eq. ([Disp-formula Ch1.E43]).

### 5.1 Bloch equations

To examine the steady-state properties of the Bloch equations, in Fig. [Fig Ch1.F5] we plot the ratios $s_{z}^{\mathrm{ss}}/s_{z}^{\mathrm{eq}}=p(\Omega ,\omega_{1})$ (first row) and $s_{x,y}^{\mathrm{ss}}/s_{z}^{\mathrm{ss}}=\pm \omega_{1}f_{x,y}(\Omega )$ (second row) against the offset frequency Ω for four different values of $B_{1}$ . A free radical with g=2 was assumed when converting $B_{1}$ to $\omega_{1}$ , so that $B_{1}=6$ G corresponds to $\omega_{1}/2\pi =16.8$ MHz. This maximum value of $B_{1}$ is intended to reflect the actual mw field of modern-day DNP spectrometers at X band [Bibr bib1.bibx21] and at J band [Bibr bib1.bibx20]. The electronic relaxation times used in the plots were $T_{2S}=60$ ns and $T_{1S}=9\;T_{2S}=540$ ns.

**Figure 5 Ch1.F5:**
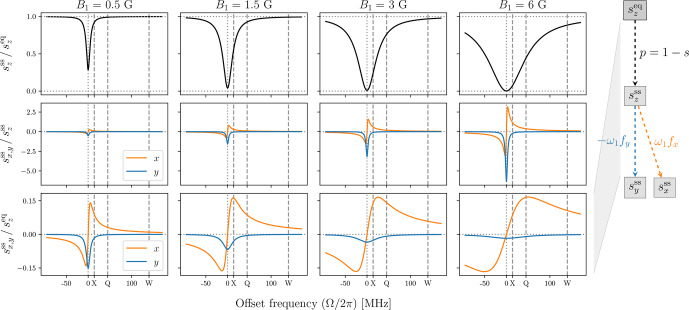
Transfer functions characterizing the steady state of the classical Bloch equations. The conversion of $B_{1}$ to $\omega_{1}$ was for free radical with g=2 ; hence $B_{1}=6$ G corresponds to $\omega_{1}/2\pi =16.8\;\mathrm{MHz}$ . In all plots $T_{2}=60$ ns and $T_{1}=9\;T_{2}$ . The positions of the nuclear Larmor frequencies at X (14 MHz), Q (45 MHz) and W (140 MHz) bands are indicated with vertical dashed lines.

From the first row of Fig. [Fig Ch1.F5] we see that the electronic saturation is most efficient on resonance ( Ω=0 ) and quickly becomes inefficient at larger offsets. With increasing mw power (different columns) the deviation of $s_{z}^{\mathrm{ss}}$ from equilibrium spreads to larger offsets. As our main interest is in the solid effect, we have indicated with dashed vertical lines the offsets Ω that correspond to proton Larmor frequencies at the X (9.2 GHz/14 MHz), Q (30 GHz/45 MHz) and W (92 GHz/140 MHz) mw bands. Considering that DNP is performed at high mw powers, let us examine the saturation at $B_{1}=6$ G (Fig. [Fig Ch1.F5], upper right plot).

Looking at $\Omega =\omega_{I}$ at X band, we see that the allowed EPR transition is almost completely saturated. Because the efficiency of the solid effect scales with p (Eq. [Disp-formula Ch1.E26]) any gain from efficiently driving the forbidden transitions will be squashed down dramatically, thus substantially reducing the ultimate enhancement of the NMR signal. This observation implies that at X band the best solid-effect enhancement may occur at less than maximum mw power, as we demonstrate numerically in Sect. [Sec Ch1.S6.SS2].

The second row of Fig. [Fig Ch1.F5] shows the offset dependence of the transfer functions connecting the longitudinal component $s_{z}^{\mathrm{ss}}$ to the transverse components $s_{x,y}^{\mathrm{ss}}$ . The observed increase in magnitude from left to right reflects the multiplication by $\omega_{1}$ of the functions $f_{x,y}$ which are independent of $\omega_{1}$ (Eq. [Disp-formula Ch1.E11]). Being the real ( $f_{y}$ ) and imaginary ( $f_{x}$ ) components of a complex-valued Lorentzian with width $R_{2S}$ and center frequency Ω=0 , these functions correspond to the absorptive and dispersive components of a homogeneous EPR line. The absorptive component (blue line) is largest at Ω=0 , while the two extrema of the dispersive component (orange line) are located at $\Omega =\pm R_{2S}$ . At offsets much larger than the locations of these extrema (i.e., $\Omega \gg R_{2S}$ ), the absorptive component drops as $1/\Omega^{2}$ , while the dispersive component drops as 1/Ω .

The third row of Fig. [Fig Ch1.F5] shows the net transfer functions relating the input of the Bloch equations, $s_{z}^{\mathrm{eq}}$ , to their ultimate outputs, $s_{x,y}^{\mathrm{ss}}$ . These transfer functions are obtained by multiplying the solid black lines in the first row by the lines in the second row. In essence, what we see are the absorptive and dispersive components of a power-broadened EPR line. The power broadening (i.e., multiplication by 1-s ) leads to qualitative differences. For example, while the peak of the blue line in the second row of the figure increased linearly with $\omega_{1}$ , it now decreases as $1/\omega_{1}$ . In the case of the orange line, the locations of its extrema are now shifted towards larger offsets ( $\Omega \approx \pm \omega_{1}(T_{1S}/T_{2S}{)}^{1/2}$ ), and their magnitude is approximately independent of $B_{1}$ ( $\approx 0.5(T_{2S}/T_{1S}{)}^{1/2}$ , which equals 1/6≈0.17 for the choice of relaxation times in Fig. [Fig Ch1.F5]). Clearly, the tail of the power-broadened dispersive (orange) component extends further into the range of interest for the solid effect at high mw frequencies than the tail of the absorptive (blue) component. One could thus expect that the path through $s_{x}^{\mathrm{ss}}$ in Fig. [Fig Ch1.F3]a (orange arrows) contributes to $v_{-}$ more than the path through $s_{y}^{\mathrm{ss}}$ (blue arrows), simply because $s_{y}^{\mathrm{ss}}$ does not survive at offsets equal to the nuclear Larmor frequencies at high fields.

**Figure 6 Ch1.F6:**
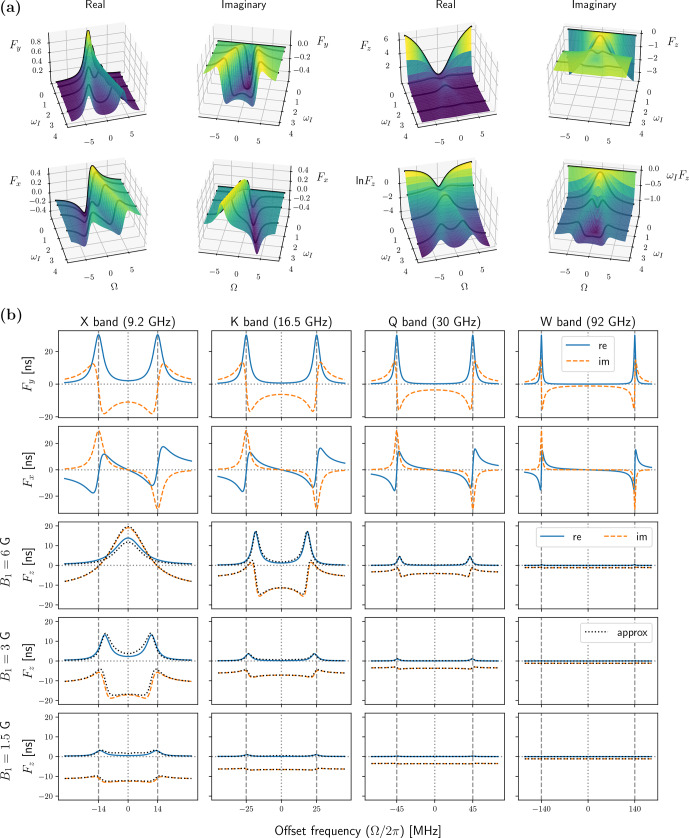
The functions $F_{y}$ , $F_{x}$ and $F_{z}$ characterizing the steady state of the second set of Bloch equations. **(a)** Angular frequencies are measured in units of $R_{2S}$ . $R_{1S}=R_{2S}/9$ as in the other figures. To calculate $F_{z}$ we used $\omega_{1}=1.5$ , which for $T_{2S}=60$ ns corresponds to $B_{1}\approx 1.5$ G. Solid black lines are cross-sections at $\omega_{I}=0$ , 0.5, 1.5 and 3. **(b)** Numerical parameters as in Fig. [Fig Ch1.F5]. Recall that $B_{1}=6$ G corresponds to $\omega_{1}/2\pi =16.8$ MHz. The approximate curves in the last three rows (dotted black) are calculated using Eq. ([Disp-formula Ch1.E51]).

### 5.2 Generalized Bloch equations

The transfer functions indicated with colored arrows in Fig. [Fig Ch1.F4]b depend on the auxiliary functions $F_{x,y}$ and $F_{z}$ (Eq. [Disp-formula Ch1.E48]). These three complex-valued functions are plotted in the $\omega_{I}$ - Ω plane in Fig. [Fig Ch1.F6]a. In the plots, the angular frequencies are reported in units of $R_{2S}$ . Cross-sections at $\omega_{I}=0$ , 0.5, 1.5 and 3 are drawn over the surfaces with solid black lines. The black lines at $\omega_{I}=0$ show that the imaginary parts of $F_{y}$ , $F_{x}$ and $F_{z}$ vanish, and their real parts become equal to $f_{y}$ , $f_{x}$ and $f_{z}$ of the classical Bloch equations (cf. Fig. [Fig Ch1.F5], first two rows). In particular, at $\omega_{I}=0$ , $F_{y}$ and $F_{x}$ as functions of Ω are like the absorptive and dispersive components of the EPR line. When plotting $F_{z}$ we used $\omega_{1}=1.5$ (in units of $R_{2S}$ ). Because both the real and imaginary parts of $F_{z}$ decay very rapidly with increasing $\omega_{I}$ , we also show the logarithm of the real part and the product of the imaginary part with $\omega_{I}$ . These transformations make the small values of $F_{z}$ at large $\omega_{I}$ visible.

In Fig. [Fig Ch1.F6]b we show these functions against Ω at four different nuclear Larmor frequencies and, in the case of $F_{z}$ , three different mw powers. In each case, the locations of the Larmor frequencies along the horizontal axis are indicated with vertical dashed lines. In the first and second rows we see $F_{y}$ and $F_{x}$ , which do not change with mw power. The real and imaginary parts of $F_{y}$ (first row) look like the real and imaginary parts of two complex-valued Lorentzians centered at $\Omega =-\omega_{I}$ and $\Omega =+\omega_{I}$ . Indeed, with

50 $$L_{\pm }={\left[R_{2S}+i\left(\omega_{I}\pm \Omega \right)\right]}^{-1},$$

it is straightforward to show that $F_{y}=(L_{-}+L_{+})/2$ . These Lorentzians have the same width as $f_{y}$ and $f_{x}$ of the classical Bloch equations (Fig. [Fig Ch1.F5], second row). The function $F_{x}$ in the second row of Fig. [Fig Ch1.F6]b also has Lorentzian-like features centered at $\Omega =\pm \omega_{I}$ , but the Lorentzian on the right is flipped around the horizontal axis. Indeed, it can be shown that $F_{x}=(L_{-}-L_{+})/2$ .

Differently from $F_{x,y}$ , $F_{z}$ depends on $\omega_{1}$ (Eq. [Disp-formula Ch1.E40]). In the last three rows of Fig. [Fig Ch1.F6]b we plot $F_{z}(\Omega )$ for three different values of $B_{1}$ , starting with $B_{1}=6$ G (third row) and going down to $B_{1}=1.5$ G (last row). The first thing to notice is that both the real (blue) and imaginary (orange) parts of this function decrease rapidly with increasing $\omega_{I}$ , i.e., moving to the right in a given row. (The former as $1/\omega_{I}^{2}$ and the latter as $1/\omega_{I}$ .) As all transfer functions in Eq. ([Disp-formula Ch1.E48]) are proportional to $F_{z}$ , we expect these to also decrease rapidly with increasing nuclear Larmor frequency.

At the lower mw powers and higher magnetic fields $F_{z}$ is seen to be dominated by its imaginary part, as its real part remains close to zero. At higher mw powers and lower magnetic fields ( $B_{1}=6$ G, X and K bands, and $B_{1}=3$ G, X band) the real and imaginary parts are seen to be comparable in magnitude. Moving from the former to the latter regime, there is a major qualitative change: the features at $\Omega =\pm \omega_{I}$ shift towards the origin ( $B_{1}=6$ G, K band, and $B_{1}=3$ G, X band) until they coalesce into a single line ( $B_{1}=6$ G, X band).

In the companion paper [Bibr bib1.bibx26], we calculate $F_{z}$ approximately using perturbation theory and find

51 $$\begin{array}{rl} F_{z} & \approx \frac{{\mathrm{cos}}^{2}\alpha }{{\tilde{R}}_{1}+i\omega_{I}}+\frac{\frac{1}{2}{\mathrm{sin}}^{2}\alpha }{{\tilde{R}}_{2}+i\left(\omega_{I}-\omega_{\mathrm{eff}}\right)}\\ & +\frac{\frac{1}{2}{\mathrm{sin}}^{2}\alpha }{{\tilde{R}}_{2}+i\left(\omega_{I}+\omega_{\mathrm{eff}}\right)}, \end{array}$$

where the frequency $\omega_{\mathrm{eff}}=(\Omega^{2}+\omega_{1}^{2}{)}^{1/2}$ corresponds to the effective magnetic field, α is the angle between this field and $B_{0}$ , such that $\mathrm{cos}\alpha =\Omega /\omega_{\mathrm{eff}}$ and $\mathrm{sin}\alpha =\omega_{1}/\omega_{\mathrm{eff}}$ , and

52 $$\begin{array}{rl} {\tilde{R}}_{1} & =R_{1S}(\mathrm{cos}\alpha {)}^{2}+R_{2S}(\mathrm{sin}\alpha {)}^{2}\\ {\tilde{R}}_{2} & =R_{2S}[1-(\mathrm{sin}\alpha {)}^{2}/2]+R_{1S}(\mathrm{sin}\alpha {)}^{2}/2. \end{array}$$

This result is exact for $R_{1S}=R_{2S}$ and is perturbative in the difference of the two electronic relaxation rates [Bibr bib1.bibx26].

The approximation in Eq. ([Disp-formula Ch1.E51]) is shown with dotted black lines in the last three rows of Fig. [Fig Ch1.F6]b. It is seen to correctly capture both the shift of the peaks towards smaller offsets and their coalescence at Ω=0 . Inspecting Eq. ([Disp-formula Ch1.E51]), we see that the dependence of $F_{z}$ on Ω comes from the second and third summands. The second summand is a complex-valued Lorentzian centered at $\omega_{\mathrm{eff}}=\omega_{I}$ , which corresponds to the offsets $\Omega =\pm (\omega_{I}^{2}-\omega_{1}^{2}{)}^{1/2}$ . This explains the deviation of the maxima from the canonical solid-effect positions $\Omega =\pm \omega_{I}$ for $\omega_{1}\approx \omega_{I}$ . At X band, when $B_{1}=6$ G, $\omega_{1}$ is larger than $\omega_{I}$ , and the two Lorentzians fuse together. It is noteworthy that the equality $\omega_{\mathrm{eff}}=\omega_{I}$ , implied by the approximation Eq. ([Disp-formula Ch1.E51]), also arises as the matching condition of the pulsed DNP method known as NOVEL (nuclear orientation via electron spin locking) [Bibr bib1.bibx16].

We now turn to the transfer functions in Eq. ([Disp-formula Ch1.E48]), which were depicted with colored arrows in Fig. [Fig Ch1.F4]b. These are plotted in the second and third rows of Fig. [Fig Ch1.F7]. As $T_{i}$ (solid red lines) is just the real part of $F_{z}$ , it exhibits all the features that we already talked about when discussing Fig. [Fig Ch1.F6]b. The dashed red lines in the third row of Fig. [Fig Ch1.F7] correspond to the mw-independent part of $T_{i}$ , namely $T_{i}^{0}=T_{i}(\omega_{1}=0)$ , which contributes to the nuclear relaxation rate $w_{1}$ rather than to $v_{+}$ (Eq. [Disp-formula Ch1.E47]). At the high mw field that we have used ( $B_{1}=6$ G), $T_{i}^{0}$ is negligible compared to $T_{i}$ (solid red line), thus subtracting the relaxation would not make much of a difference. However, at lower mw powers the contribution of $T_{i}$ to thermal relaxation becomes comparable to the rest, and the correction makes a difference. (This can be seen in the bottom plot of Fig. [Fig App1.Ch1.S1.F11], where $B_{1}=1$ G.)

**Figure 7 Ch1.F7:**
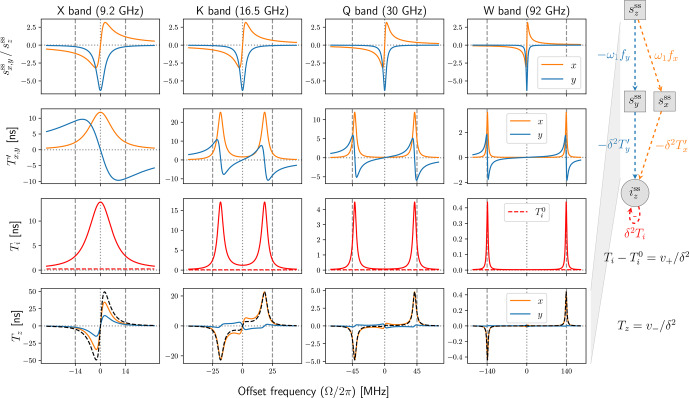
Transfer functions characterizing the steady state of the two coupled sets of Bloch equations. Used parameters: $B_{1}=6$ G, $T_{2S}=60$ ns and $T_{1S}=9\;T_{2S}$ .

In the second row of Fig. [Fig Ch1.F7], the functions $T_{x,y}^{\prime }$ resulted from the product of $F_{y,x}$ and $F_{z}$ (Eq. [Disp-formula Ch1.E48]). Interestingly, their Lorentzian-like features are at the same frequency offsets as those of $T_{i}$ , the real part of $F_{z}$ . We observe that $T_{x}^{\prime }$ (orange) and $T_{y}^{\prime }$ (blue) are similar in magnitude. Thus, if $s_{x}^{\mathrm{ss}}$ and $s_{y}^{\mathrm{ss}}$ were comparable in magnitude, the contributions of the two parallel branches from $s_{z}^{\mathrm{ss}}$ to $i_{z}^{\mathrm{ss}}$ would be similar (see flow diagram in the right margin of Fig. [Fig Ch1.F7]). We know, however, that $s_{y}^{\mathrm{ss}}$ is much smaller than $s_{x}^{\mathrm{ss}}$ at large offsets (Fig. [Fig Ch1.F7], first row), and so the path via $T_{x}^{\prime }$ (orange) will contribute more.

Multiplying the functions $T_{x,y}^{\prime }$ (Fig. [Fig Ch1.F7], second row) by the functions in the first row, we obtain the orange and blue lines in the last row of the figure. (The functions in the first row were shown before in Fig. [Fig Ch1.F5]. They are plotted here again only for $B_{1}=6$ G. The four plots are identical to each other but appear different due to the different scales of the horizontal axes.) Comparing the first and second rows of Fig. [Fig Ch1.F7], we see that an odd/even function in the first row is multiplied by an even/odd function in the second row to produce the corresponding orange and blue lines in the bottom row. As a result, the contribution of both parallel paths from $s_{z}^{\mathrm{ss}}$ to $i_{z}^{\mathrm{ss}}$ (via either $s_{x}^{\mathrm{ss}}$ or $s_{y}^{\mathrm{ss}}$ ) is odd in Ω . The cumulative transfer function of the two parallel paths (Eq. [Disp-formula Ch1.E49]) is also plotted in the last row of Fig. [Fig Ch1.F7] with dashed black lines. At Q and W bands it is seen to be essentially identical to its first additive contribution $\omega_{1}f_{x}T_{x}$ (orange line), which means that the electronic polarization is transferred to the nucleus almost entirely through the dispersive component $s_{x}^{\mathrm{ss}}$ .

In the light of this observation, we will now rewrite the cumulative transfer function $T_{z}$ (Eq. [Disp-formula Ch1.E49]) as if the polarization was transferred only through the dispersive component. We start by observing that

53 $$F_{y}f_{x}+F_{x}f_{y}=F_{y}\frac{2R_{2S}+i\omega_{I}}{R_{2S}+i\omega_{I}}f_{x}=F_{y}^{\prime }f_{x},$$

where the last equality defines $F_{y}^{\prime }$ . The second $R_{2S}$ in the numerator of Eq. ([Disp-formula Ch1.E53]) comes from $F_{x}f_{y}$ and can be viewed as a “correction” to $F_{y}f_{x}$ due to $F_{x}f_{y}$ . Introducing

54 $$T_{x}=\text{Re}\left\{iF_{z}\left(\omega_{1}F_{y}^{\prime }\right)\right\}$$

(compare this $T_{x}$ with $T_{x}^{\prime }$ in Eq. [Disp-formula Ch1.E48]), we rewrite Eq. ([Disp-formula Ch1.E42]) in a way that contains $s_{x}^{\mathrm{ss}}$ but does not contain $s_{y}^{\mathrm{ss}}$ as follows:

55 $${\dot{i}}_{z}{|}_{\mathrm{coh}}^{\mathrm{ss}}=-\left(\delta^{2}T_{i}\right)i_{z}^{\mathrm{ss}}-\left(\delta^{2}T_{x}\right)s_{x}^{\mathrm{ss}}.$$

Note that this expression is exact and does not result from simply dropping the last term in Eq. ([Disp-formula Ch1.E42]), which is proportional to $s_{y}^{\mathrm{ss}}$ , as the contribution of the path through $s_{y}^{\mathrm{ss}}$ is taken into account in the definition of $T_{x}$ .

Equation ([Disp-formula Ch1.E55]) is depicted in Fig. [Fig Ch1.F4]d, which shows only one path from $s_{z}^{\mathrm{ss}}$ to $i_{z}^{\mathrm{ss}}$ going through $s_{x}^{\mathrm{ss}}$ . From Fig. [Fig Ch1.F4]d,

56 $$v_{-}=\left(\omega_{1}f_{x}\right)\left(\delta^{2}T_{x}\right).$$

This factorization is revisited in Sect. [Sec Ch1.S7.SS1].

## 6 Closer look at the rate constants

### 6.1 Relation to the classical rates

Here we show that the classical expression of the ZQ and DQ transition rates (Eq. [Disp-formula Ch1.E28]) follows from the exact rates (Eqs. [Disp-formula Ch1.E47] and [Disp-formula Ch1.E56]) when $\omega_{1}\ll \omega_{I}$ . To simplify the analysis, we take from the start a long electronic $T_{1}$ relaxation time, such that $R_{1S}\ll \omega_{I}$ . This should be the case for high-field DNP in solids, where the electronic $T_{1}$ is at least a microsecond. In this case, the function $F_{z}$ (Eq. [Disp-formula Ch1.E40]) simplifies to

57 $$F_{z}\approx \frac{1}{i\omega_{I}+\omega_{1}^{2}F_{y}}=\frac{1}{i\omega_{I}}{\left(1+\frac{\omega_{1}^{2}}{i\omega_{I}}F_{y}\right)}^{-1}.$$

For $\omega_{1}\ll \omega_{I}$ , to first order in $\omega_{1}^{2}$ ,

58 $$F_{z}\approx \frac{1}{i\omega_{I}}+\frac{\omega_{1}^{2}}{\omega_{I}^{2}}F_{y}.$$

Note that, because the relaxation rate $R_{1S}$ was neglected, $T_{i}^{0}=\text{Re}\{F_{z}(\omega_{1}=0)\}=0$ . In other words, the contribution of the short path in Fig. [Fig Ch1.F3]b (blue and red arrow) to the nuclear relaxation rate vanishes. From Eqs. ([Disp-formula Ch1.E47]) and ([Disp-formula Ch1.E56]), retaining only terms of up to first order in $\omega_{1}^{2}$ ,

59 $$v_{+}\approx \delta^{2}\frac{\omega_{1}^{2}}{\omega_{I}^{2}}\;\text{Re}\{F_{y}\},\;v_{-}\approx \delta^{2}\frac{\omega_{1}^{2}}{\omega_{I}^{2}}\;\omega_{I}f_{x}\text{Re}\left\{F_{y}\frac{2R_{2S}+i\omega_{I}}{R_{2S}+i\omega_{I}}\right\}.$$

To establish the equivalence of these expressions with Eq. ([Disp-formula Ch1.E28]), we need to show that $\text{Re}\{F_{y}\}$ and $\omega_{I}f_{x}\text{Re}\{F_{y}^{\prime }\}$ equal, respectively, the sum and difference of two real-valued Lorentzians centered at $\Omega =\pm \omega_{I}$ . For the complex-valued Lorentzians in Eq. ([Disp-formula Ch1.E50]), we already observed that $L_{-}+L_{+}=2F_{y}$ . One can also confirm that $\text{Re}\{L_{-}-L_{+}\}=2\omega_{I}f_{x}\text{Re}\{F_{y}^{\prime }\}$ . Hence,

60 $$v_{\pm }\approx \delta^{2}\frac{\omega_{1}^{2}}{\omega_{I}^{2}}\;\frac{1}{2}\left(\text{Re}\{L_{-}\}\pm \text{Re}\{L_{+}\}\right),$$

and thus

61 $$v_{0,2}\approx \frac{1}{8}\left(A_{1}^{*}A_{1}\right)\frac{\omega_{1}^{2}}{\omega_{I}^{2}}\text{Re}\{L_{\pm }\},$$

which is equivalent to the classical expression (Eq. [Disp-formula Ch1.E28]).

The sum and difference of the classical rates $v_{2}$ and $v_{0}$ are compared with the exact $v_{\pm }$ in the first two rows of Fig. [Fig Ch1.F8]. Naturally, the Lorentzians associated with the classical rates remain centered at $\pm \omega_{I}$ even when the maxima of the exact rates shift closer to each other at Q and K bands and converge at X band. At high fields (e.g. W band), where $\omega_{I}\gg \omega_{1}$ , the classical approximations work perfectly.

In the last row of Fig. [Fig Ch1.F8] we show the DQ-transition rate $v_{2}$ . While, classically, it is always non-negative (dashed black lines), the exact rate deduced from $v_{\pm }$ (solid brown lines) is seen to become negative at some offsets. From the perspective of the rate-equation formalism, such negative rates are meaningless. In that sense, the description of the forbidden transitions in terms of $v_{\pm }$ is more fundamental than their description in terms of $v_{0}$ and $v_{2}$ .

**Figure 8 Ch1.F8:**
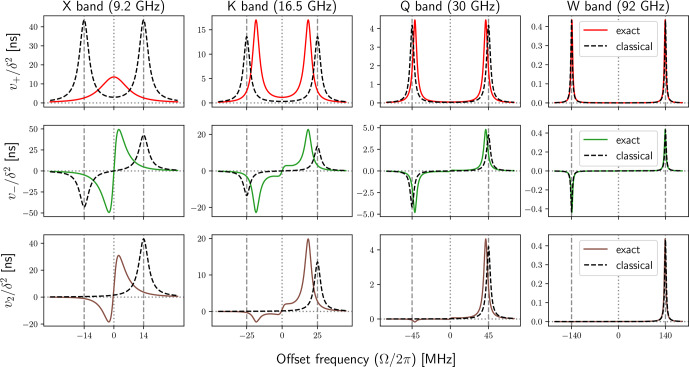
Forbidden-transition rates calculated either exactly (solid lines) or using the classical expression (Eq. [Disp-formula Ch1.E28]) with $v_{\pm }=v_{2}\pm v_{0}$ (dashed lines). As in the previous figures, $B_{1}=6$ G, $T_{2S}=60$ ns and $T_{1S}=9\;T_{2S}$ .

**Figure 9 Ch1.F9:**
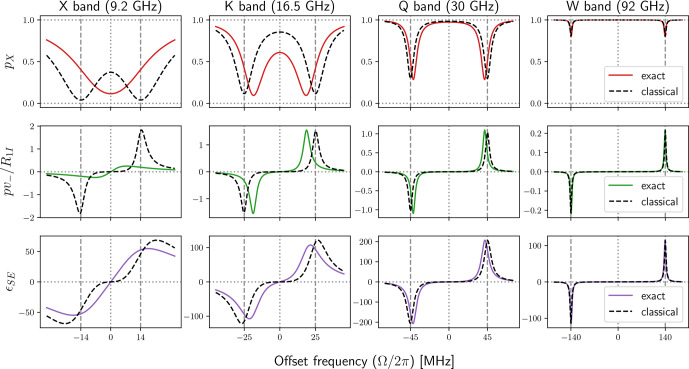
Decomposition of the DNP field profile ( $\epsilon_{\mathrm{SE}}$ ) in terms of the multiplicative contributions $p_{X}$ and $pv_{-}/R_{1I}$ . The new parameters used here are $T_{1I}=30$ ms, b=1 nm and N=0.1 M. Other parameters: $B_{1}=6$ G, $T_{2S}=60$ ns and $T_{1S}=9\;T_{2S}$ .

### 6.2 Solid-effect DNP enhancement

The DNP enhancement of the solid effect (Eq. [Disp-formula Ch1.E26]) can be written as the product of $|\gamma_{S}|/\gamma_{I}$ with the following two dimensionless factors:

62 $$p_{X}=\frac{R_{1I}/\delta^{2}}{R_{1I}/\delta^{2}+\left(T_{i}-T_{i}^{0}\right)},\;\frac{pv_{-}}{R_{1I}}=\frac{pT_{z}}{R_{1I}/\delta^{2}},$$

which we have rewritten here in terms of the transfer functions $T_{i}$ , $T_{i}^{0}$ and $T_{z}$ . These transfer functions already appeared in the last two rows of Fig. [Fig Ch1.F7]. Thus, to calculate the DNP enhancement, we only need to specify the ratio $R_{1I}/\delta^{2}$ .

In the case of δ , rather than calculating $A_{1}$ (Eq. [Disp-formula Ch1.E30]) for some arbitrary inter-spin vector, let us average $A_{1}^{*}A_{1}$ over the entire 3D space. With b denoting the so-called “distance of closest approach” or “contact distance”, and N denoting the number of electron spins per unit volume, we have

63 $$\langle \delta^{2}\rangle =\frac{1}{4}\langle A_{1}^{*}A_{1}\rangle =D_{\mathrm{dip}}^{2}\frac{6\pi }{5}\frac{N}{3b^{3}},$$

where, in this case, the angular brackets denote spatial averaging. We will use b=1 nm and N=0.1 M as representative but otherwise arbitrary values.

While the average over 3D space in Eq. ([Disp-formula Ch1.E63]) is clear mathematically, it is important to understand that physically it implies fast spin diffusion [Bibr bib1.bibx34]. Since the nuclear polarization in solids is homogenized across the sample through spin diffusion, replacing the individual $\delta^{2}$ 's of the nuclear spins by the average over all nuclei is only legitimate when spin diffusion is faster than the nuclear spin-lattice relaxation. In practice, spin diffusion is rather slow and is often the bottleneck for efficient polarization transfer in solids [Bibr bib1.bibx17]. As a result, the DNP enhancement values that we will calculate with Eq. ([Disp-formula Ch1.E63]) are expected to be appreciably larger than what could be observed experimentally.

Similar considerations also apply for the choice of the nuclear spin-lattice relaxation time. In principle $T_{1I}$ will depend on the distance of the nucleus from the electronic spin and thus will vary greatly across the sample. In the limit of fast spin diffusion, however, only its average value becomes relevant. In general, this time depends on the radical concentration and on the magnetic field $B_{0}$ . However, for the purposes of illustration, here we take a generic numerical value of $T_{1I}=30$ ms across all mw bands. Again, this value is realistic but otherwise arbitrary.

Using b=1 nm, N=0.1 M and $T_{1I}=30$ ms we find $R_{1I}/\langle \delta^{2}\rangle =1.78$ ns. Let us visually compare this timescale with $(T_{i}-T_{i}^{0})=v_{+}/\langle \delta^{2}\rangle$ by consulting the solid red line in the first row of Fig. [Fig Ch1.F8]. We observe that at X and K bands the maxima of the red line are much larger than 2 ns, which means that the minima of $p_{X}$ will be close to zero. At Q band the maxima of the red line are comparable to 2 ns, and at W band they are much smaller. The minima of the nuclear cross-polarization factor are thus expected to be about 1/2 and 1, respectively. These expectations are confirmed by the maroon lines in the first row of Fig. [Fig Ch1.F9] and demonstrate that the ratio $p_{X}$ can substantially deviate from one at lower magnetic fields.

To estimate the expected magnitude of the second factor in Eq. ([Disp-formula Ch1.E62]), we need to compare the timescale $R_{1I}/\langle \delta^{2}\rangle =1.78$ ns with $pT_{z}$ . While $T_{z}$ was shown with dashed black lines in the bottom row of Fig. [Fig Ch1.F7], now it has to be multiplied by the electronic polarization factor in the top row of Fig. [Fig Ch1.F5]. From the line for $B_{1}=6$ G in this row, we see that $T_{z}$ will be significantly suppressed at X band, so it is hard to judge how the reduced value will compare with 1.78 ns. At Q band, $T_{z}$ will be reduced by a little more than a factor of 2, which will make its peak in Fig. [Fig Ch1.F7] comparable to $R_{1I}/\langle \delta^{2}\rangle$ . At W band, where the factor p is about 0.9, $T_{z}$ will be only slightly reduced, so its peak is expected to be about one-fifth of 1.78 ns. Again, these estimates are confirmed by the green lines in the second row of Fig. [Fig Ch1.F9].

The last row of Fig. [Fig Ch1.F9] shows the product of the first two rows times $|\gamma_{S}|/\gamma_{I}$ , assuming a proton spin. The result is the solid-effect DNP enhancement (Eq. [Disp-formula Ch1.E26]). In the figure we have also shown the factors predicted by the classical expression of the rates (Eq. [Disp-formula Ch1.E28]) with dashed black lines. While there are quantitative differences between the exact calculations and the classical approximation, the magnitudes of the DNP enhancements in the two cases are, in fact, comparable. A closer look reveals that, for the specific $B_{1}$ and relaxation times used in the calculations, the classical description of the solid effect (Eq. [Disp-formula Ch1.E28]) works perfectly at Q band and at larger mw frequencies. (In Fig. [Fig App1.Ch1.S1.F12] we show that by reducing the mw power to $B_{1}=1$ G the classical expressions are also perfect at X band.) The amplitudes of the maximum enhancements at the four mw bands are roughly in the ratios 1 : 2 : 4 : 2 (X : K : Q : W). On the other hand, considering the inverse dependence on $\omega_{I}^{2}$ , we expect the ratios 100 : 40 : 10 : 1. These expected ratios are indeed observed at the much lower mw power of $B_{1}=1$ G (Fig. [Fig App1.Ch1.S1.F12]b). Comparison of Figs. [Fig Ch1.F9] and [Fig App1.Ch1.S1.F12] shows that increasing $B_{1}$ increases the amplitudes of the maximum enhancements at W and Q bands but reduces the enhancement at X band. Such reduction of the solid-effect DNP enhancement with increasing $B_{1}$ has been reported at X band [Bibr bib1.bibx21].

## 7 Concluding discussion

### 7.1 Refactorization of the polarization transfer

When $p_{X}\approx 1$ (Eq. [Disp-formula Ch1.E26]), e.g., at high magnetic fields (Fig. [Fig Ch1.F9], W band) and lower mw powers (Fig. [Fig App1.Ch1.S1.F12]b), the DNP enhancement of the solid effect is

64 $$\epsilon_{\mathrm{SE}}\approx \left(pv_{-}\right)T_{1I}|\gamma_{S}|/\gamma_{I}\;\left(p_{X}\approx 1\right).$$

Since $T_{1I}$ is easily accessible experimentally, $pv_{-}$ is the only non-trivial factor in Eq. ([Disp-formula Ch1.E64]). From Fig. [Fig Ch1.F4]a we know that p relates $s_{z}$ at steady state to $s_{z}^{\mathrm{eq}}$ , and from Fig. [Fig Ch1.F4]c we know that $v_{-}$ relates the time derivative of $i_{z}$ at steady state to $s_{z}$ . Hence, the product $pv_{-}$ relates the time derivative of $i_{z}$ directly to the electronic Boltzmann polarization $s_{z}^{\mathrm{eq}}$ , as shown graphically in Fig. [Fig Ch1.F10]a.

Since, by construction, the rate equations of the polarizations do not model the dynamics of the coherences, their steady state balances the rates of mw excitation only against the longitudinal (i.e., spin-lattice) relaxations. The polarization factor p quantifies this balance for the allowed EPR transition (Eq. [Disp-formula Ch1.E3]). Because the rate equations work only with the polarizations, all dynamical variables between $s_{z}$ and $i_{z}$ in Fig. [Fig Ch1.F2]d are lumped into the rate constant $v_{-}$ . Classically, this rate constant ( $v_{-}=v_{2}-v_{0}$ ) is obtained by calculating the rates of the ZQ ( $v_{0}$ ) and DQ ( $v_{2}$ ) transitions using first-order perturbation theory. From this point of view, decomposing the product $pv_{-}$ into the factors p and $v_{-}$ is natural. The offset dependence of these two factors was visualized in Fig. [Fig Ch1.F5] (top row) and Fig. [Fig Ch1.F8] (middle row, dashed black lines). The curves for $B_{1}=6$ G and W band are reproduced in Fig. [Fig Ch1.F10]a (black and green lines).

**Figure 10 Ch1.F10:**
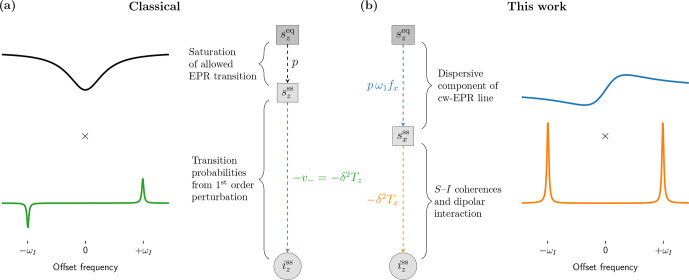
Two ways of decomposing the effect of the electronic Boltzmann polarization ( $s_{z}^{\mathrm{eq}}$ ) on the steady-state nuclear polarization ( $i_{z}^{\mathrm{ss}}$ ). The classical expression **(a)** partitions this effect into the factors p and $v_{-}=v_{2}-v_{0}$ , which reflect respectively the saturation of the allowed EPR transition and the excitation of the forbidden DQ ( $v_{2}$ ) and ZQ ( $v_{0}$ ) transitions. Alternatively **(b)**, the same effect can be written as the product of the dispersive component of the power-broadened EPR line ( $s_{x}^{\mathrm{ss}}/s_{z}^{\mathrm{eq}}$ ) and the rate constant $\delta^{2}T_{x}$ . The latter characterizes the steady state of the electron–nucleus coherences without any contribution from the purely electronic coherences.

In contrast to this classical approach, here we considered the complete spin dynamics of relevance to the solid effect, including the dynamics of the coherences (Fig. [Fig Ch1.F2]d). The analysis was simplified by the realistic assumption that the electronic dynamics was not affected by the dipolar interaction with the nuclear spins. Thus, in our description, the purely electronic degrees of freedom constitute an isolated dynamical system, which influences the other dynamical variables but is not affected by them.

This division of the complete dynamical system into a purely electronic part and the rest calls for a similar separation of the product $pv_{-}$ in Eq. ([Disp-formula Ch1.E64]) into an electronic part and a mixed electron–nucleus part. Such factorization of $pv_{-}$ is illustrated in Fig. [Fig Ch1.F10]b, where the purely electronic part is identified with the dispersive component of the EPR line. This would be the out-of-phase cw-EPR spectrum recorded under the same mw power as used in the DNP experiment. Then, from Eq. ([Disp-formula Ch1.E56]), the second factor is recognized to be $\delta^{2}T_{x}$ , where $\delta^{2}$ accounts for the strength of the dipolar interaction (Eq. [Disp-formula Ch1.E41]), and $T_{x}$ takes care of the interconnections between the relevant electron–nucleus coherences at steady state (Eq. [Disp-formula Ch1.E54]). The offset dependence of the dispersive EPR line was visualized before in Fig. [Fig Ch1.F5] (bottom row). The curve for $B_{1}=6$ G is reproduced in Fig. [Fig Ch1.F10]b (blue line). The curve below it (orange line) corresponds to $\delta^{2}T_{x}$ at W band, which is essentially the same as $\delta^{2}T_{x}^{\prime }$ that was shown in the second row of Fig. [Fig Ch1.F7] since at this high magnetic field $T_{y}^{\prime }$ contributes negligibly little.

Because, as already illustrated above (Fig. [Fig Ch1.F9], middle row, W band), the classical approach and our new approach lead to essentially the same product $pv_{-}$ , the new factorization in Fig. [Fig Ch1.F10]b may appear as a purely mathematical exercise of little practical interest. Note, however, that recognizing the dispersive EPR line as contributing multiplicatively to the DNP enhancement suggests that the dispersive extrema could become visible in the field profile of the enhancement, provided that they are not fully suppressed by the factor $\delta^{2}T_{x}$ . Such possibility is completely missing in the classical description on the left-hand side of Fig. [Fig Ch1.F10], where any reference to the dispersive EPR line and its extrema is irrelevant.

In the companion paper [Bibr bib1.bibx26], we show that, in liquids, the random modulation of the dipolar interaction broadens the lines of the factor $\delta^{2}T_{x}$ (Fig. [Fig Ch1.F10]b, orange line). When the tails of these broadened lines reach the extrema of the dispersive EPR line (blue line), the enhancement field profile exhibits features that are reminiscent of the DNP effect known as thermal mixing [Bibr bib1.bibx20]. These features are a direct manifestation of the dispersive EPR line in the DNP spectrum [Bibr bib1.bibx26].

### 7.2 Origin of the solid effect

The issue of *Comptes rendus* from 9 April 1958 contained the article “*Effect of nuclear polarization in liquids and gases adsorbed on charcoal*” by Erb, Motchane and Uebersfeld [Bibr bib1.bibx13]. It reported enhancements of the proton NMR signal of benzene upon mw irradiation of the EPR line of charcoal. The enhancements were positive at fields larger than the EPR resonance position and negative at smaller fields. Because fields symmetrically displaced from the resonance yielded the same magnification factor, the enhancement profile was odd in the field offset and resembled the dispersive component of the EPR line. The similarity between the two prompted the authors to augment the Solomon equation [Bibr bib1.bibx31] with two new terms proportional to $s_{x}$ and $s_{y}$ [Bibr bib1.bibx13]:

65 $${\dot{i}}_{z}=\lambda \left(i_{z}-i_{z}^{\mathrm{eq}}\right)+\mu \left(s_{z}-s_{z}^{\mathrm{eq}}\right)+\nu s_{x}+\rho s_{y}.$$

Taking into account that “under saturation conditions $s_{y}=0$ ”, the authors arrived at

66 $${\dot{i}}_{z}=\lambda \left(i_{z}-i_{z}^{\mathrm{eq}}\right)+\mu \left(s_{z}-s_{z}^{\mathrm{eq}}\right)+\nu s_{x}.$$

Assuming μ was small in their case, they solved Eq. ([Disp-formula Ch1.E66]) at steady state as

67 $$i_{z}^{\mathrm{ss}}=i_{z}^{\mathrm{eq}}-\left(\nu /\lambda \right)s_{x}^{\mathrm{ss}},$$

which explained the similarity between the field profile of the enhancement and the dispersive EPR line.

Intriguingly, with μ=0 , the phenomenological equation (Eq. [Disp-formula Ch1.E65]) is mathematically identical to Eq. ([Disp-formula Ch1.E42]), which expressed the time derivative of $i_{z}$ at steady state as a linear combination of $i_{z}$ , $s_{x}$ and $s_{y}$ . The argument of [Bibr bib1.bibx13] that the contribution of $s_{y}$ could be neglected, which let to Eq. ([Disp-formula Ch1.E66]), is justified by our analysis. Specifically, in the last row of Fig. [Fig Ch1.F7] we observed that the contribution of the absorptive component $s_{y}$ to the rate constant $v_{-}$ was smaller than that of the dispersive component $s_{x}$ . Moreover, we showed that Eq. ([Disp-formula Ch1.E55]) was, in fact, exact within the framework of our treatment. Thus, the phenomenological equation (Eq. [Disp-formula Ch1.E66]) produces the correct steady state when its coefficients are selected as $\nu =-\delta^{2}T_{x}$ and $\lambda =-\delta^{2}(T_{i}-T_{i}^{0})$ .

The next installment of *Comptes rendus* from 14 April 1958 contained Abragam and Proctor's report “*A new method for dynamic polarization of atomic nuclei in solids*” [Bibr bib1.bibx4], which was printed 132 pages after [Bibr bib1.bibx13]. This seminal contribution provided the modern theoretical understanding, and subsequently also the name, of the solid-state effect of dynamic nuclear polarization. In particular, the authors argued that the excitation of the forbidden transitions (++)⇌(--) and (+-)⇌(-+) , which become weakly allowed because the dipolar coupling yields mixed states of the form (--)+q(-+) , could be used for DNP. ( ± are the states of the two spin types, both taken as 1/2 for simplicity.) As an experimental verification of the theoretical proposal, the Boltzmann polarization of ${}^{19}$ F nuclei was used to enhance the NMR signal of ${}^{6}$ Li in a LiF monocrystal, thus demonstrating polarization transfer from nuclei with larger to nuclei with smaller gyromagnetic ratios (i.e., a *nuclear* solid effect).

One month and a half after Abragam and Proctor's report, in the 28 May 1958 issue of *Comptes rendus*, Erb, Motchane and Uebersfeld published another report with the lengthy title “*On a new method of nuclear polarization in fluids adsorbed on charcoal. Extension to solids and in particular to irradiated organic substances*” [Bibr bib1.bibx14]. There, the authors state the following (our translation):

The experiments [Bibr bib1.bibx13] had been carried out with charcoal whose half-linewidth was 5 gauss and the multiplication factor seemed to reproduce the paramagnetic dispersion curve.

The new experiments … indicated that the increase in polarization of the proton in the adsorbed fluid is maximum in all cases, when the electronic and nuclear frequencies are chosen such that the nuclear resonance field differs from the electron resonance field δH=±5 gauss (within 10 %).

These results support the suggestion of Abragam that the new theory of Abragam and Proctor on the nuclear polarization in solids [Bibr bib1.bibx4] must apply to these new phenomena, and invalidates the interpretation proposed previously [Bibr bib1.bibx13].

The value of 5 gauss found in the case of the proton indeed corresponds to the value deduced from the theoretical formula $H_{0}\pm \delta H=(\omega \pm \omega_{N})/\gamma_{e}$ ,… .

This seems to have sealed the fate of the insightful observation of [Bibr bib1.bibx13] that the odd parity of the solid-effect DNP field profile resembles the dispersive component of the EPR line.

With the understanding developed in the 65 years since these first publications on the solid effect, the additional transverse terms in Eq. ([Disp-formula Ch1.E65]) appear strange and even disturbing. Nevertheless, our analysis showed that in one specific regime, namely at steady state, Eq. ([Disp-formula Ch1.E65]) is exact. Admittedly, because of the algebraic relationships between all dynamical variables at steady state, the transverse components in Eq. ([Disp-formula Ch1.E65]) can be expressed in terms of the longitudinal component, as we did when going from Eq. ([Disp-formula Ch1.E42]) to Eq. ([Disp-formula Ch1.E43]). Such mathematical manipulation, however, only highlights the fact that the value of any description of spin dynamics by rate equations, independently of whether it contains transverse components or not, lies in the proper selection of the phenomenological rate constants. In this paper, we departed from the classical approach of identifying these rate constants with the transition probabilities per unit time. Instead, completely disregarding the dynamical aspect of the rate equations, we selected the phenomenological rate constants by requiring that the steady state of the exact quantum dynamics is correctly reproduced.

By writing the rate equation of the nuclear polarization with explicit dispersive component (Eq. [Disp-formula Ch1.E66]), [Bibr bib1.bibx13] reached the conclusion that the DNP enhancement depends *multiplicatively* on $s_{x}$ (Eq. [Disp-formula Ch1.E67]). This conclusion is confirmed by our analysis. Indeed, from the new perspective illustrated in Fig. [Fig Ch1.F10]b, the DNP field profile acquires its odd parity in Ω directly from the dispersive component of the EPR line (blue line), exactly as intuited by [Bibr bib1.bibx13]. Certainly, one could explain the odd parity of the solid-effect DNP enhancement in various other ways that do not involve the dispersive EPR line, as has been done in the past 65 years. The validity of these other explanations, however, does not invalidate the intuition of Erb, Motchane, and Uebersfeld.

## 8 Conclusions

In this paper we developed a novel way of thinking about the solid effect, which was grounded in the dynamics of the spins at steady state. The main insight of our dynamical description relates to the role of the coherences.

While our analysis focused on the solid effect and the Hamiltonian in Eq. ([Disp-formula Ch1.E29]), the systematic procedure for deriving the relevant equations of motion under a given spin Hamiltonian (Sect. [Sec Ch1.S3.SS2]), and the developed graphical representations to visualize the interplay of these equations (Sect. [Sec Ch1.S4]) and their steady state (Sect. [Sec Ch1.S5]), should be applicable to other related effects with different Hamiltonians.

The classical explanation of the solid effect in terms of state mixing [Bibr bib1.bibx4] is static in nature and is thus hard to generalize to liquids where the dipolar interaction fluctuates randomly due to molecular motions. The time-dependent description of the solid effect developed here naturally accommodates such stochastic modulation of the parameters of the Hamiltonian, in a way similar to the treatment of relaxation in liquids chap. VIII. In the companion paper [Bibr bib1.bibx26], the formalism is extended to the solid effect in liquids, and its predictions are validated against recent DNP experiments at J band [Bibr bib1.bibx20].

## Figures and Tables

**Figure A1 App1.Ch1.S1.F11:**
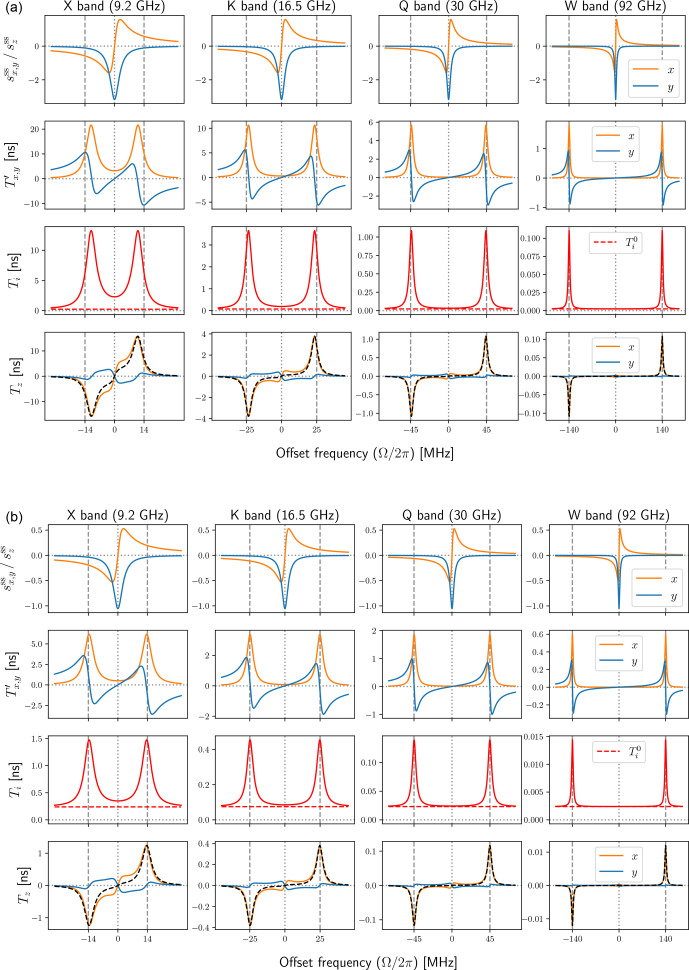
Same as Fig. [Fig Ch1.F7] with smaller mw fields of $B_{1}=3$ G **(a)** and $B_{1}=1$ G **(b)**.

**Figure A2 App1.Ch1.S1.F12:**
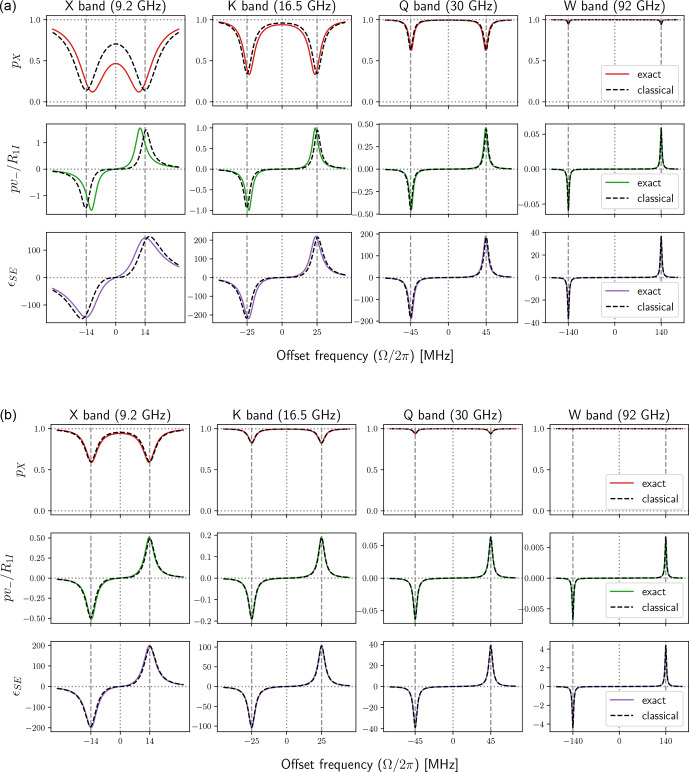
Same as Fig. [Fig Ch1.F9] with smaller mw fields of $B_{1}=3$ G **(a)** and $B_{1}=1$ G **(b)**.

## Data Availability

No data sets were used in this article.

## Appendix A Additional figures

The numerical examples in the paper were for the excessively high mw field of $B_{1}=6$ G, which is reachable with a custom-designed resonance structure [Bibr bib1.bibx11]. As the modern-day DNP experiments in solids are generally performed without a mw resonator, here we show numerical examples for the lower fields of $B_{1}=3$ and $B_{1}=1$ G. Although these are still likely an order of magnitude larger than what is used in practice, the figures aim to illustrate how some of the features discussed in the paper progressively change upon reduction of $B_{1}$ .

## Code availability

The code used to generate the figures is at https://github.com/dzsezer/solidDNPliquids (https://doi.org/10.5281/zenodo.7990757, Sezer, 2023b).
